# Metformin rejuvenates Nap1l2‐impaired immunomodulation of bone marrow mesenchymal stem cells via metabolic reprogramming

**DOI:** 10.1111/cpr.13612

**Published:** 2024-02-13

**Authors:** Fan Liu, Ruohui Han, Shaochen Nie, Yuxin Cao, Xinming Zhang, Feng Gao, Zhengyang Wang, Liangyu Xing, Zhaoguang Ouyang, Lei Sui, Wenyi Mi, Xudong Wu, Lu Sun, Meilin Hu, Dayong Liu

**Affiliations:** ^1^ Department of Endodontics and Laboratory of Stem Cells Endocrine Immunology Tianjin Medical University School of Stomatology Tianjin China; ^2^ Department of Prosthodontics Tianjin Medical University School of Stomatology Tianjin China; ^3^ Tianjin Institute of Immunology, The Province and Ministry Co‐sponsored Collaborative Innovation Center for Medical Epigenetics, Key Laboratory of Immune Microenvironment and Disease of the Ministry of Education Tianjin Medical University General Hospital, Tianjin Medical University Tianjin China; ^4^ State Key Laboratory of Experimental Hematology, The Province and Ministry Co‐sponsored Collaborative Innovation Center for Medical Epigenetics, Key Laboratory of Immune Microenvironment and Disease (Ministry of Education), Department of Cell Biology Tianjin Medical University Tianjin China; ^5^ Department of Periodontics and Oral Medicine University of Michigan School of Dentistry Ann Arbor Michigan USA; ^6^ Periodontal and Implant Microsurgery Academy (PiMA) University of Michigan School of Dentistry Ann Arbor Michigan USA

## Abstract

Ageing and cell senescence of mesenchymal stem cells (MSCs) limited their immunomodulation properties and therapeutic application. We previously reported that nucleosome assembly protein 1‐like 2 (Nap1l2) contributes to MSCs senescence and osteogenic differentiation. Here, we sought to evaluate whether Nap1l2 impairs the immunomodulatory properties of MSCs and find a way to rescue the deficient properties. We demonstrated that metformin could rescue the impaired migration properties and T cell regulation properties of OE‐Nap1l2 BMSCs. Moreover, metformin could improve the impaired therapeutic efficacy of OE‐Nap1l2 BMSCs in the treatment of colitis and experimental autoimmune encephalomyelitis in mice. Mechanistically, metformin was capable of upregulating the activation of AMPK, synthesis of l‐arginine and expression of inducible nitric oxide synthase in OE‐Nap1l2 BMSCs, leading to an increasing level of nitric oxide. This study indicated that Nap1l2 negatively regulated the immunomodulatory properties of BMSCs and that the impaired functions could be rescued by metformin pretreatment via metabolic reprogramming. This strategy might serve as a practical therapeutic option to rescue impaired MSCs functions for further application.

## Graphical abstract

In this study, we demonstrated that overexpression of Nap1l2 impaired the immunomodulation capacities of BMSCs through reduced NO production. Interestingly, metformin could rescue the impaired immunomodulatory ability of OE‐Nap1l2 BMSCs both in vitro and in vivo by activating the AMPK signalling pathway.

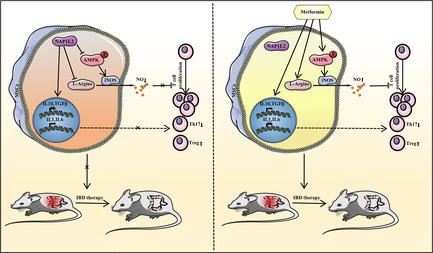

## 1 INTRODUCTION

Growing evidence has indicated that mesenchymal stem cells (MSCs) exhibit immunomodulatory capability, which could regulate immune responses and participate in tissue repair.^1^, ^2^, ^3^ Preclinical studies and clinical trials of MSCs transplantation are widely performed to treat a variety of autoimmune and inflammatory diseases, including systemic lupus erythematosus,^4^ multiple sclerosis,^5^ inflammatory bowel disease,^6^ Sjögren syndrome,^7^ as well as chronic periodontitis.^8^, ^9^, ^10^, ^11^, ^12^ When MSCs are exposed to inflammatory stimuli, they can be recruited locally to the sites of damaged tissues and further produce various chemokines (CCL5 and CXCL9), growth factors (TGF‐β, HGF, etc.) and immune‐regulatory molecules (IL‐10, nitric oxide and PGE2), thereby regulating immune homeostasis.^8^, ^9^, ^10^, ^11^, ^12^ When activated by inflammatory cytokines, MSCs were shown to skew the CD4+ T‐helper population phenotypes, suppress T cell proliferation and cytokine production respectively.^13^, ^14^, ^15^ One prominent candidate in the underlying mechanisms of mouse MSC‐mediated immunosuppression is nitric oxide (NO), which could be catalysed by inducible nitric oxide synthase (iNOS) in response to inflammatory cytokines.^16^ Meanwhile, the immunosuppressive effect and therapeutic effect in MSCs with iNOS deletion are diminished.^17^

Ageing was reported to affect the immunomodulatory ability of MSCs and limit their therapeutic effects by regulating telomere shortening, DNA damage, epigenetic modification changes, and production of senescence‐associated secretory phenotype (SASP).^18^, ^19^, ^20^ Our previous study had indicated that overexpression of nucleosome assembly protein 1‐like 2 (Nap1l2) of bone marrow MSCs was associated with cell senescence and impaired osteogenesis by downregulation of histone 3 lysine 4 acetylation and activation of the NF–κB signalling pathway.^21^ However, the effect and underlying mechanism of Nap1l2 on the immunomodulatory capability of bone marrow MSCs remain poorly understood.

AMPK is a key regulator of many cellular pathways that are linked to health span and lifespan. The sensitivity of AMPK declines with ageing, which leads to the secretion of pro‐inflammatory SASP factors by stimulating the p38/MAPK and NF–κB pathways. Meanwhile, metformin, an activator of AMPK, has been reported to delay ageing by impacting dysregulated nutrient sensing, mitochondrial dysfunction, epigenetic modification, stem cell exhaustion and cell senescence.^22^, ^23^, ^24^ Metformin could inhibit the SASP and cell senescence by activating AMPK.^25^ In addition, metformin delays stem cell senescence and rejuvenates its multiple differentiation capacities by activating AMPK and inhibiting mTORC1 to regulate autophagy.^26^ However, whether and how metformin regulates the immunomodulation capacity of MSCs also needs to be further investigated.

In this study, we demonstrated that the migration and immunomodulation capacities of mouse bone marrow mesenchymal stem cells (BMSCs) were impaired after overexpression of Nap1l2. Besides, when BMSCs were transplanted in mice with experimental inflammatory bowel disease (IBD) or experimental autoimmune encephalomyelitis (EAE), the therapeutic efficacy of BMSCs was significantly decreased after Nap1l2 overexpression. Interestingly, metformin could rescue the impaired immunomodulatory ability of OE‐Nap1l2 BMSCs both in vitro and in vivo. The underlying mechanisms were investigated by analysing metabolomics, l‐arginine‐iNOS‐nitric oxide and AMPK pathways.

## 2 METHODS

### 2.1 Animals and cell isolation

Male C57BL/6 mice (Beisitong, China) were used for the animal experiments and all mice experiments were performed with the approval of the Animal Welfare and Ethical Committee (IRM‐DWLL‐2022113).

Bone marrow stem cells were isolated from femur and tibia marrow compartments and were purified by the characteristics of differential attachment.^27^ BMSCs were cultured using Dulbecco's modified Eagle's medium (DMEM) including 15% fetal bovine serum (FBS) (Gibco, Life Technologies, USA) and 100 U/mL penicillin‐100 μg/mL streptomycin. On day 3, the culture medium was replaced to remove non‐adherent cells. The medium was subsequently changed for 3 days, and the MSCs were used within four passages. A single‐cell suspension of mouse splenocytes was obtained by crushing spleens through a 100 μm cell strainer and red blood cells were lysed in the cell suspensions. Splenocytes were then cultured in RPMI‐1640 medium supplemented with 100 U/mL penicillin‐100 μg/mL streptomycin and 10% fetal bovine serum.

### 2.2 Lentivirus preparation and transduction

Both the enforced overexpression and suppression of NAP1L2 were achieved by lentivirus‐carrying vectors. Plasmid construction and virus collection were performed as described previously.^21^ BMSCs were plated overnight and then infected with lentiviruses in the presence of polybrene (8 μg/mL; Sigma‐Aldrich, USA) for 12 h. Cells were then selected using puromycin for 48 h and resistant clones were pooled. Real‐time qPCR was used to assess the efficiencies of knockdown and overexpression.

### 2.3 In vitro wound healing assay

For the wound healing assay, BMSCs were seeded on 6‐well plates and cultured to reach 100% confluency. The cell monolayers were scratched with a sterile 200 μL pipette tip and maintained in media without serum. Images of the scratched area were observed under a microscope and captured at 0, 24 and 48 h after the scratch was created.

### 2.4 Cell migration assay

Cells (2 × 10^5^ cells/well) were seeded onto the upper chamber of 8 μm Transwell plates (Corning, USA) and cultured in 100 μL media without serum, whereas medium containing 15% serum was added to the lower chamber. After 24 h incubation, cells remaining on the upper surface were removed, the migrating cells were stained with crystal violet. At least three wells were counted per experiment.

### 2.5 Primary murine splenocyte proliferation assessment

To analyse splenocyte proliferation, cells (1 × 10^6^/sample) were incubated with 5 mM of carboxyfluorescein succinimidyl ester (CFSE, Invitrogen) for 10 min in a 37°C water bath. Then five volumes of ice‐cold medium were added to stop the staining reaction and the cells were washed three times with the final culture medium. Splenocytes were cocultured with BMSCs for 72 h with CD3/CD28 beads (Gibco, Life Technologies, USA) and IL‐2 (BioLegend, USA), then harvested for CFSE flow cytometry analysis.

### 2.6 Flow cytometry assay

BMSCs (2 × 10^5^ cells/well) were plated in triplicate in 12‐well plates for 1 day; then, cells (1 × 10^6^ cells/well) from the spleen were cocultured with stem cells. After cocultured, FITC anti‐CD4 and PE anti‐CD25 were used for surface staining, whereas Alexa Fluor 647 anti‐FOXP3 antibodies were used for intracellular staining. To detect T helper 17 (Th17) cells, the spleen cells were stimulated with a cell stimulation cocktail (Tonbo Biosciences, USA) for 6 h. Then cells were incubated with FITC anti‐CD8a and PerCP anti‐CD3 for 30 min. After being fixed and permeabilized, cells were stained with PE anti‐IL‐17A (BioLegend, USA). All data were collected by flow cytometry and analysed using FlowJo software (Tree Star, USA). The antibodies and drugs used in this study are listed in Table S1.

### 2.7 Quantitative real‐time PCR analysis

Total RNA was extracted with TRIzol reagent (Life Technologies, USA) followed by two‐step quantitative RT–PCR analysis of RNA transcripts with HiScript III RT SuperMix for qPCR (Vazyme, China) and Universal SYBR qPCR Master Mix (Vazyme, China) according to the manufacturer's instructions. Target gene expression was normalized to the expression level of GAPDH. The primers for specific genes are displayed in Table S2.

### 2.8 Western blot analysis

Cells were lysed with RIPA buffer containing phosphatase inhibitor cocktail on ice before centrifuging at 4°C for 5 min, and the protein concentration of each sample was determined by BCA Protein Assay (Thermo Fisher Scientific, USA). Equal amounts of cell lysates were mixed with SDS loading buffer and loaded for immunoblot. All gels were subjected to PVDF membrane transfer before blocking with TBST buffer containing 5% BSA for 2 h. Then incubated overnight with primary antibodies with gentle shaking. After being washed by TBST buffer, the membranes were incubated with secondary antibodies for 60 min at room temperature. Images were acquired using an ECL solution. The antibodies used in this study are listed in Table S1.

### 2.9 Concentration measurement of nitric oxide

The supernatant of BMSCs was collected after stimulated with TNF‐α (20 ng/mL) and IFN‐γ (20 ng/mL). The concentration of NO was assessed to determine nitrate and nitrite levels by using a Griess reagent (Beyotime, China).

### 2.10 Metabolomics analysis of BMSCs

The cells were washed with PBS three times at 37°C, collected into a centrifuge tube, and immediately frozen in liquid nitrogen. A total of 800 μL of cold mixed solution (methanol: acetonitrile = 1: 1) was added to extract the metabolites. The mixture was centrifuged at 14,000 g for 5 min to collect the supernatant and the supernatant was dried in a vacuum centrifuge for a non‐targeted metabolomics test. For LC–MS analysis, the samples were re‐dissolved in 100 μL acetonitrile/water (1:1, v/v) solvent. For quality control (QC) analysis, samples were prepared by pooling 10 μL of each sample and analysed together with the other samples. Metabolite structure identification and data processing were extracted by XCMS software after raw MS data were converted to MzXML files. The processed data were analysed by multivariate data analysis, including Pareto‐scaled principal component analysis (PCA), screening of significantly changed metabolites, correlation analysis of different metabolites and KEGG pathway analysis.

### 2.11 Experimental IBD mouse model

To induce acute colitis, 3% DSS (molecular mass 36–40 kDa, MP Biomedicals, USA) in drinking water was provided ad libitum for 7 days. On days 1, 4 post‐DSS induction, 2 × 10^5^ stem cells were administered in IBD mice through the tail vein injection. All mice were euthanized on day 7 for tissue analysis.

The disease activity index (DAI), including body weight, stool consistency and hidden blood, was assessed daily to evaluate the severity of colitis as previously described.^28^ Hidden blood of stools was assessed using the Hemoccult II test (Beckman Coulter, Canada).

Intact colons from the epityphlon to the anus were harvested, and colon lengths were measured immediately after photographs of the colon were acquired. Formalin‐fixed paraffin‐embedded (FFPE) colon tissues were sectioned for histopathology staining with haematoxylin and eosin (H&E). The histological activity index (HAI) score was adopted to evaluate the degree of epithelial damage and mucosal inflammation infiltration of colon tissues as previously described.^29^

### 2.12 Statistical analysis

Statistical tests were carried out using Student's *t‐*test, one‐way analysis of variance or two‐way analysis of variance with GraphPad Prism. The statistical significance between the two independent groups was compared by the Student's *t*‐test. Comparisons of three or more independent groups were performed by using the one‐way analysis of variance. Differences in disease activity index between groups at different times were determined using a two‐way analysis of variance. The significant difference compared with control is indicated as follows: *p < 0.05, **p < 0.01, and ***p < 0.001.

## 3 RESULTS

### 3.1 Nap1l2 impaired immunomodulatory properties of BMSCs derived from mice

To understand the function of Nap1l2 on BMSCs, we used lentivirus carrying shRNA or vector expressing mouse Nap1l2 to knock down or overexpress Nap1l2 (Figure S1A). Scratch assay and transwell migration assay showed that the migration ability of OE‐Nap1l2 BMSCs was significantly limited, while knockdown of Nap1l2 enhanced the migration ability (Figure S1B, C).

Previous studies suggested that MSCs could produce soluble factors (NO, TGF‐β, IL‐1, IL‐6 and IL‐10) to achieve anti‐inflammatory properties, including influencing the proliferation and polarization of CD4+ T cells.^30^, ^31^, ^32^ We therefore investigated the expression of inflammatory factors in BMSCs. In the OE‐Nap1l2 group, Il1 and Il6 were obviously upregulated, whereas the expressions of Tgfβ and Il10 were decreased (Figure S1D).

**FIGURE 1:**
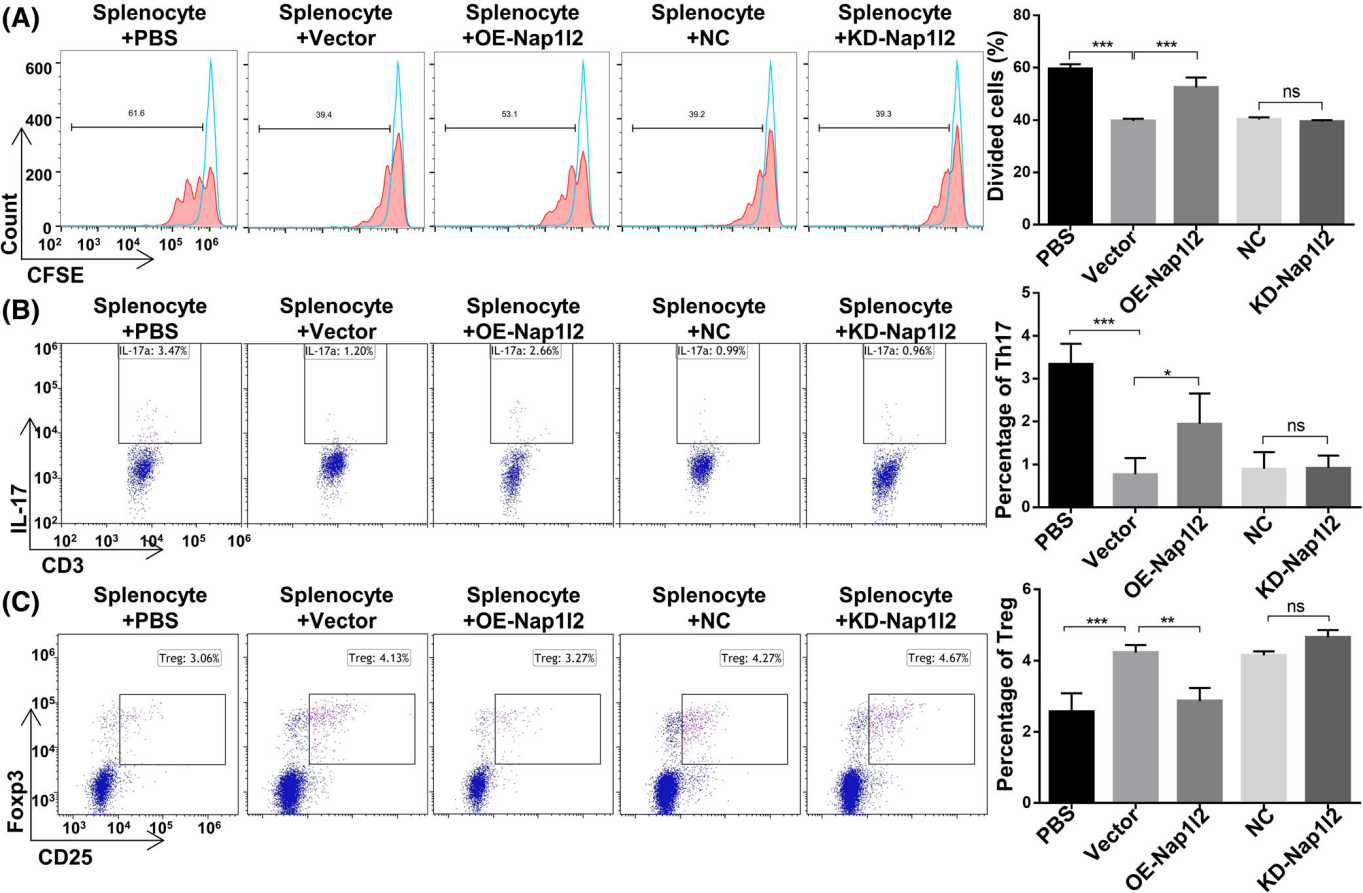
Nap1l2 impared the T cell regulation capacities of BMSCs. (A) Flow cytometry analysis showing the proliferation of CD4+ T cells that were cocultured with BMSCs. (B) Flow cytometry analysis showing the percentage of CD3 + CD8 − IL17+ cells in splenocytes (Spl) cocultured with BMSCs after Nap1l2 overexpression or knockdown. (C) Flow cytometry analysis showing the percentage of CD4 + CD25 + Foxp3+ cells in splenocytes cocultured with BMSCs after Nap1l2 overexpression or knockdown. Statistical significance was determined by one‐way ANOVA. Data were presented as mean ± SD (*n* ≥ 3). *p < 0.05, **p < 0.01, ***p < 0.001; ns, not significance.

Next, we separated splenocytes (Spl) and cocultured them with overexpression‐Nap1l2 BMSCs or Nap1l2‐knckdown BMSCs to investigate whether Nap1l2 could affect the proliferation and polarization of T cells in vitro. We found that the CD4+ T lymphocyte proliferation was significantly reduced when splenocytes were cocultured with vector, NC and KD‐Nap1l2 BMSCs, but not when cocultured with OE‐Nap1l2 BMSCs (Figures 1A and S2A). After cocultured with BMSCs, the number of CD3 + CD8 − IL17+ T cells (Th17 cells) was decreased, whereas the number of CD4 + CD25 + Foxp3+ T cells (Treg cells) was increased (Figures 1B, C and S2B, C). However, the proportion of Th17 cells was significantly raised and Treg cells were decreased after cocultured with OE‐Nap1l2 BMSCs compared to coculturing with vector BMSCs (Figures 1B, C and S2B,C). There was no significant difference after cocultured with the NC group or KD‐Nap1l2 group.

### 3.2 Overexpression of Nap1l2 reduced the therapeutic effect of BMSCs in IBD and EAE mice

**FIGURE 2:**
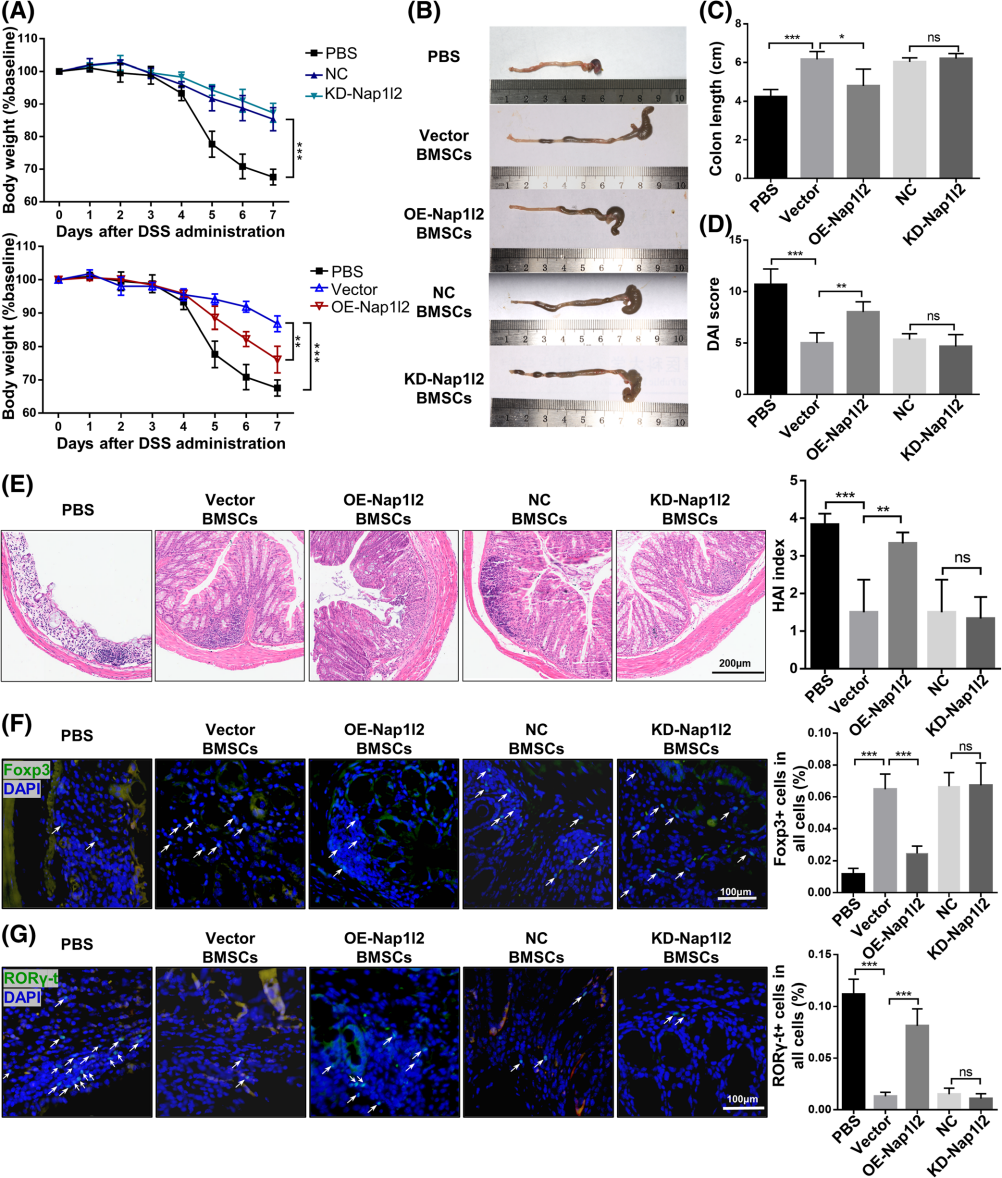
Manipulation of Nap1l2 expression altered the therapeutic efficiency of BMSCs in DSS‐induced IBD. Mice received 3% DSS dissolved in drinking water for 7 days. (A) Body weight, (B, C) colon length and (D) disease activity index (DAI) of healthy mice and IBD mice treated with PBS, vector BMSCs, OE‐Nap1l2 BMSCs, NC BMSCs and KD‐Nap1l2 BMSCs on days 1 and 4 post‐IBD induction. (E) Histology activity index (HAI) score and photomicrographs of an H&E‐stained paraffin section of colon. Scale bar, 200 μm. Levels of (F) Foxp3+ cells and (G) RORγ‐t + cells in situ (*white triangles*) were illustrated in immunofluorescent staining. Scale bar, 100 μm. Statistical significance was determined by one‐way ANOVA or two‐way ANOVA. Data were presented as mean ± SD (*n* ≥ 3). *p < 0.05, **p < 0.01, ***p < 0.001; ns, not significance.

As Nap1l2 overexpression was demonstrated to inhibit the migration and anti‐inflammation properties of BMSCs in vitro, we then sought to investigate whether Nap1l2 overexpression could affect the anti‐inflammatory effect of BMSCs in vivo. We applied BMSCs to treat dextran sulfate sodium‐induced colitis and found that vector, NC and KD‐Nap1l2 BMSCs were effective in treating IBD. However, compared with the vector group, OE‐Nap1l2 BMSCs treatment could not effectively rescue the IBD, including body weight loss, colon length and DAI score (Figure 2A–D). The expressions of pro‐inflammatory cytokines Tnf‐α and Il17 in the colon were significantly decreased in groups injected with BMSCs transduced with empty vector, but not in the colons in groups injected with OE‐Nap1l2 BMSCs (Figure S3A,B). H&E staining further demonstrated increased mucosal erosion, vacuolar hydropic degeneration of cells, increased inflammatory infiltration and necrosis in the mice treated with OE‐Nap1l2 BMSCs compared with vector BMSCs (Figure 2E). Compared with vector BMSCs, injection of OE‐Nap1l2 BMSCs locally decreased the number of Tregs and increased the number of Th17 cells (Figure 2F,G).

To investigate the impact of Nap1l2 on MSC in the treatment of autoimmune diseases in the nervous system, we constructed an EAE animal model. For EAE mice, caudal vein injection of BMSCs was managed on days 9, 11 and 13. We observed that OE‐Nap1l2 BMSCs were less effective in reducing inflammation, resulting in higher disease scores compared with vector BMSCs (Figure S4A). Histology showed that mice treated with OE‐Nap1l2 BMSCs had large inflammatory demyelinated lesions in the spinal cords (Figure S4B). Most importantly, Nap1l2 overexpression could weaken the efficiency of BMSCs for treating inflammatory diseases.

### 3.3 Manipulation of Nap1l2 expression altered the metabolism of BMSCs

Next, to explore the mechanisms by which Nap1l2 overexpression impaired the immunomodulatory properties of BMSCs, we analysed whether Nap1l2 overexpression affected the metabolism of OE‐Nap1l2 BMSCs. Partial least squares discrimination analysis (OPLS–DA) revealed that the model was stable and viable (Figure S5A). The permutation plot indicated that the original OPLS–DA model was valid and had no overfitting (Figure S5B). All metabolites were classified by chemical taxonomy and differential metabolites were visualized by the volcano plot (Figures S5E–F and 3A). It indicated that glycolysis/gluconeogenesis, fatty acid metabolism, and amino acid metabolism were changed after the overexpression of Nap1l2 (Figure S6A–C).

**FIGURE 3:**
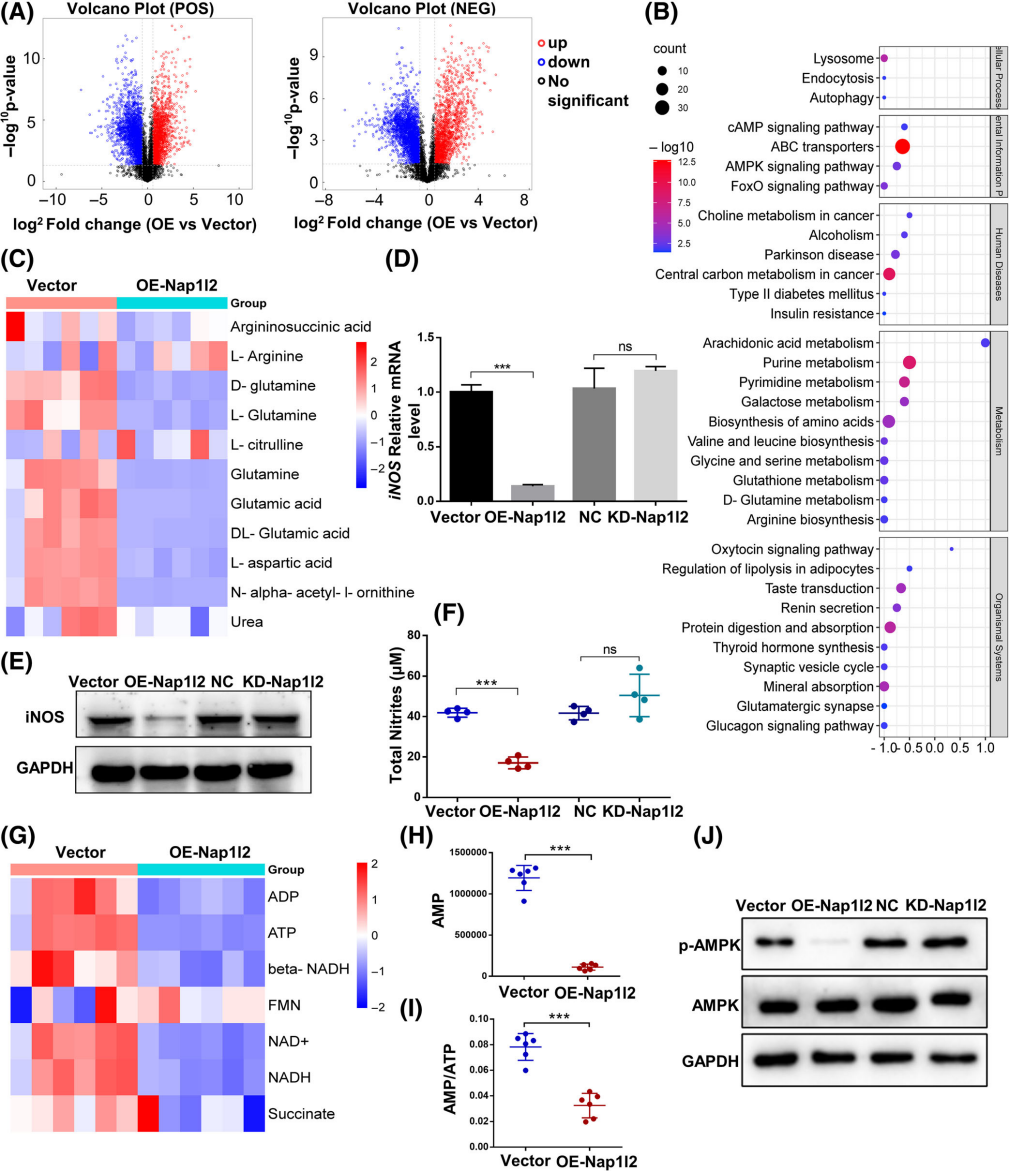
High expression of Nap1l2 altered the metabolism of BMSCs. (A) The metabolite level signature of BMSCs with overexpression of Nap1l2 and differential metabolites with fold change >1.5 or <0.67, *p* < 0.05 were visualized in the volcano plot. (B) KEGG pathway analysis of vector BMSCs and OE‐Nap1l2 BMSCs. (C) Heat map of l‐arginine biosynthesis metabolism of vector BMSCs and OE‐Nap1l2 BMSCs. (D) Expression levels of iNOS mRNA in TNF‐α and IFN‐γ (20 μg/mL each) treated BMSCs after Nap1l2 overexpression or knockdown. (E) Protein levels of iNOS in BMSCs treated with TNF‐α, IFN‐γ after Nap1l2 overexpression or knockdown. (F) Griess reagent assaying for nitrate from the supernatant of BMSCs after stimulated by TNF‐α and IFN‐γ (20 μg/mL each) for 24 h. (G) Heat map of oxidative phosphorylation metabolism of vector BMSCs and OE‐Nap1l2 BMSCs. (H) The level of AMP in vector BMSCs and OE‐Nap1l2 BMSCs. (I) The AMP:ATP ratio of vector BMSCs and OE‐Nap1l2 BMSCs. (J) Protein expression levels of p‐AMPK and AMPK of BMSCs. Statistical significance was determined by Student's *t*‐test or one‐way ANOVA. Data were presented as mean ± SD (*n* ≥ 3). *p < 0.05, **p < 0.01, ***p < 0.001; ns, not significance.

Under the stimulation of inflammatory factors such as IFN‐r and TNF‐α, BMSCs highly expressed iNOS to produce NO for regulating immune cells.^33^ As l‐arginine is the catalytic substrate for iNOS, we analysed the level of l‐arginine in BMSCs. The heat map showed that arginine synthesis was also affected after overexpression Nap1l2 (Figure 3B,C). Western blot and RT‐PCR detected significantly lower expression of iNOS in OE‐Nap1l2 BMSCs under the stimulation of IFN‐r and TNF‐α compared to the control group (Figure 3D,E). Griess test detected a low generation of NO in BMSCs with overexpressed Nap1l2 (Figure 3F). Therefore, it could be speculated that Nap1l2 overexpression affected the immunomodulation property of BMSCs by decreasing NO production.

Moreover, the oxidative phosphorylation level of OE‐Nap1l2 BMSCs was markedly changed with a decreased AMP/ATP ratio (Figure 3G–I). KEGG pathway analysis suggested that the decreased AMP/ATP ratio is closely associated with the AMPK signalling pathway (Figure 3B). Western blot demonstrated that overexpression of Nap1l2 suppressed the phosphorylation level of AMPK (Figure 3J).

To confirm the role of Nap1l2, we established a replicative cellular senescence model in the BMSCs. Accordingly, we analysed BMSCs at passage 4 (P4), and passage 16 (P16) by SA‐β‐GAL staining. A significant increase in β‐galactosidase‐positive senescent cells was observed from P4 to P16 (Figure S7A,B). Simultaneously, the expression mRNA profile of the senescence‐related genes p21 and p16 was remarkably elevated from P4 to P16 (Figure S7C). After the knockdown of Nap1l2, the expression of iNOS and production of NO was augmented in BMSCs at P16 compared to the NC group (Figure S7D–F). Western blot demonstrated that the AMPK phosphorylation level significantly increased while knockdown of Nap1l2, indicating that manipulation of Nap1l2 could affect the activation of the AMPK signalling pathway (Figure S7G).

### 3.4 Metformin regulated the metabolism of OE‐Nap1l2 BMSCs by AMPK/iNOS signalling pathway

**FIGURE 4:**
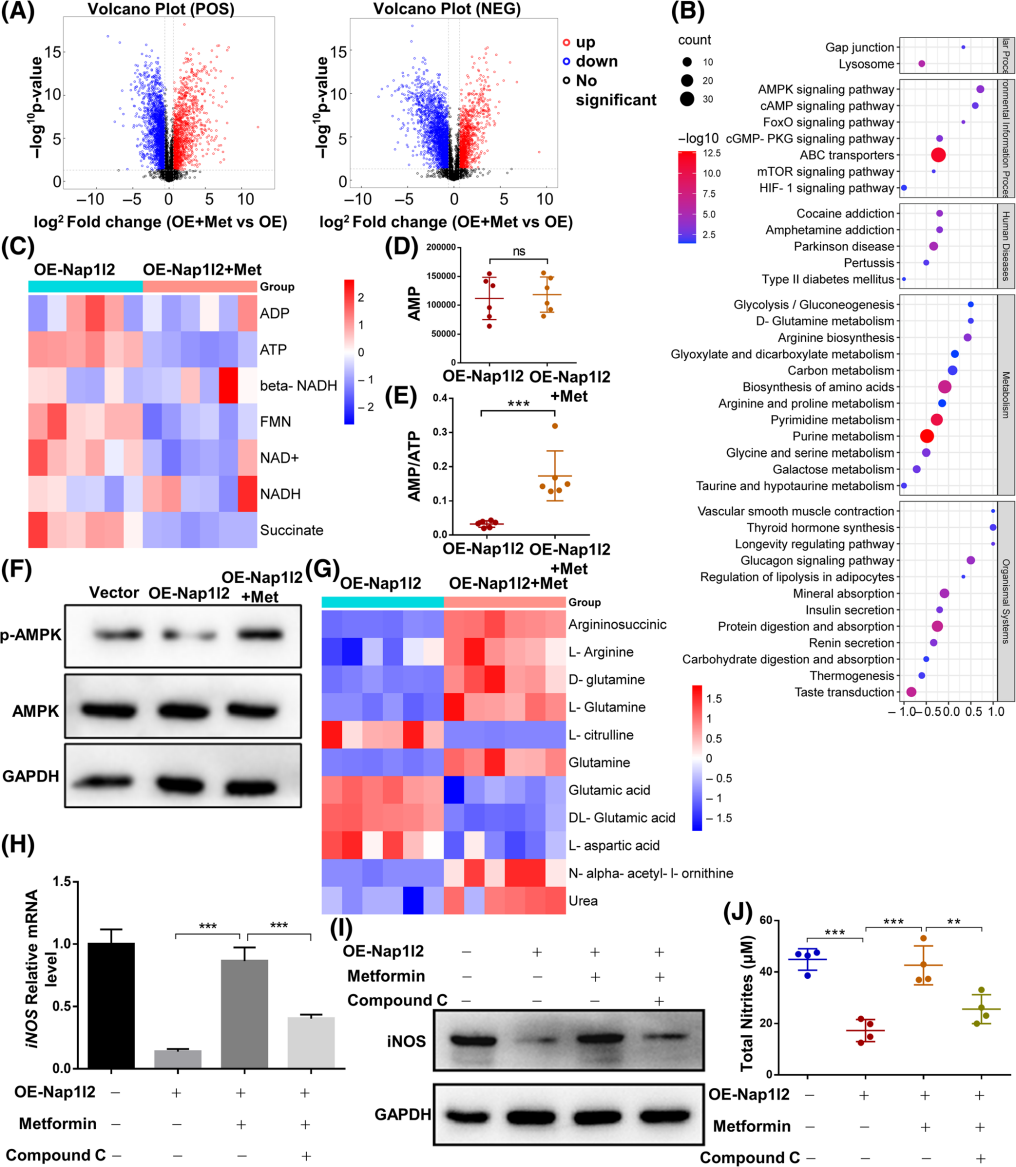
The metabolism of OE‐Nap1l2 BMSCs after using metformin. (A) Volcano plot showing differential metabolites in the OE‐Nap1l2 BMSCs after metformin treatment (fold change ≥1.5, *p* < 0.05, *n* = 6). (B) The KEGG pathway analysis of OE‐Nap1l2 BMSCs and metformin‐treated OE‐Nap1l2 BMSCs. (C) Heat map of oxidative phosphorylation metabolism of OE‐Nap1l2 BMSCs after metformin treatment. (D) The level of AMP in OE‐Nap1l2 BMSCs and metformin‐treated OE‐Nap1l2 BMSCs. (E) The AMP/ATP ratio of OE‐Nap1l2 BMSCs and metformin‐treated OE‐Nap1l2 BMSCs. (F) Protein expression levels of p‐AMPK and AMPK of OE‐Nap1l2 BMSCs after metformin treatment. (G) Heat map of arginine biosynthesis metabolism of OE‐Nap1l2 BMSCs and OE‐Nap1l2 BMSCs with metformin. (H) Expression levels of iNOS mRNA in OE‐Nap1l2 BMSCs treated with TNF‐α, IFN‐γ.(I) Protein levels of iNOS in OE‐Nap1l2 BMSCs treated with TNF‐α, IFN‐γ. (J) Griess reagent assay to determine nitrate from the supernatant of OE‐Nap1l2 BMSCs after stimulated by TNF‐α and IFN‐γ (20 μg/mL each) for 24 h. Statistical significance was determined by Student's *t*‐test or one‐way ANOVA. Data were presented as mean ± SD (*n* ≥ 3). *p < 0.05, **p < 0.01, ***p < 0.001; ns, not significance.

Metformin could regulate cellular homeostasis such as proliferation, energy metabolism and protein synthesis through activating AMPK by increasing AMP/ATP and ADP/ATP ratios.^34^, ^35^ We next considered using metformin to rescue the immunomodulation properties of OE‐Nap1l2 BMSCs. OPLS–DA analysis showed that the model was valid and had no overfitting (Figure S5C, D). All metabolites were classified by chemical taxonomy, and differential metabolites were visualized by volcano plot (Figure 4A). It showed that glycolysis/gluconeogenesis, fatty acid metabolism, and amino acid metabolism were changed after using metformin treatment (Figure S6D–F). The KEGG pathway showed that metformin could activate the AMPK signalling pathway (Figure 4B). After stimulation with metformin, oxidative phosphorylation was suppressed, causing an increasing AMP/ATP ratio in OE‐Nap1l2 BMSCs (Figure 4C–E). We observed from the western blot that metformin could influence the expression of p‐AMPK and thus activate the AMPK signalling pathway (Figure 4F).

Metformin could promote the l‐arginine biosynthesis thus increasing the level of l‐arginine (Figure 4G). Besides, metformin promoted iNOS expression and NO synthesis under the stimulation with IFN‐r and TNF‐α (Figure 4H–J). To illustrate metformin could activate the AMPK signalling pathway by regulating iNOS expression, we utilized compound C to inhibit the AMPK signalling pathway. Western blot detected decreased expression levels of iNOS after using compound C (Figure 4J). Collectively, these data indicated that metformin was able to regulate iNOS expression through the AMPK signalling pathway.

### 3.5 Metformin activated the migration of OE‐Nap1l2 BMSCs and the regulation of immune cells

**FIGURE 5:**
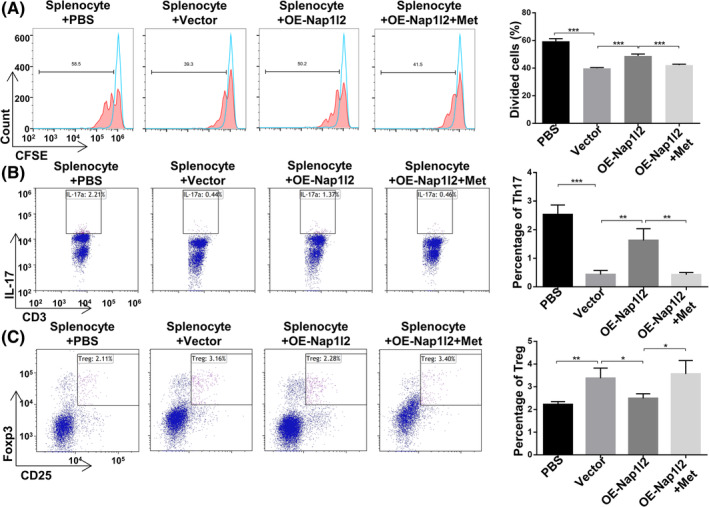
The effects of metformin on OE‐Nap1l2 BMSCs in T cell subsets regulation. (A) Flow cytometric analysis showing the proliferation of CD4+ T cells that were cocultured with BMSCs. (B) Flow cytometric analysis showing the percentage of CD3 + CD8 − IL17+ cells in splenocytes cocultured with BMSCs treated with metformin. (C) Flow cytometry analysis showing the percentage of CD4 + CD25 + Foxp3+ cells in splenocytes cocultured with BMSCs treated with metformin. Statistical significance was determined by one‐way ANOVA. Data were presented as mean ± SD (*n* ≥ 3). *p < 0.05, **p < 0.01, ***p < 0.001; ns, not significance.

We therefore sought to examine whether metformin could rescue cell migration and inflammatory mediator production in OE‐Nap1l2 BMSCs. Scratch assay and transwell test revealed that cell migration was significantly promoted in OE‐Nap1l2 BMSCs after treatment with metformin (Figure S8A,B). The secretion of inflammatory factors was also greatly altered with Il6 and Il1 expression downregulated and Tgfβ and Il10 expression upregulated in a dose‐dependent manner (Figure S8C). When metformin‐treated BMSCs were cocultured with splenocytes, CD4+ T cell proliferation was inhibited compared with OE‐Nap1l2 BMSCs (Figures 5A and S9A). Compared to the OE‐Nap1l2 BMSCs groups, the metformin‐treated group exhibited a lower enrichment of Th17 cells and a higher proportion of Treg cells (Figures 5B,C and S9B,C). These data indicated that metformin improved the ability of OE‐Nap1l2 BMSCs to regulate T‐cell polarization and proliferation in vitro. This effect may be caused by increased NO production and altered expression of inflammatory factors.

### 3.6 Metformin treatment ameliorated the therapeutic effect of OE‐Nap1l2 BMSCs in autoimmune diseases in mouse models

**FIGURE 6:**
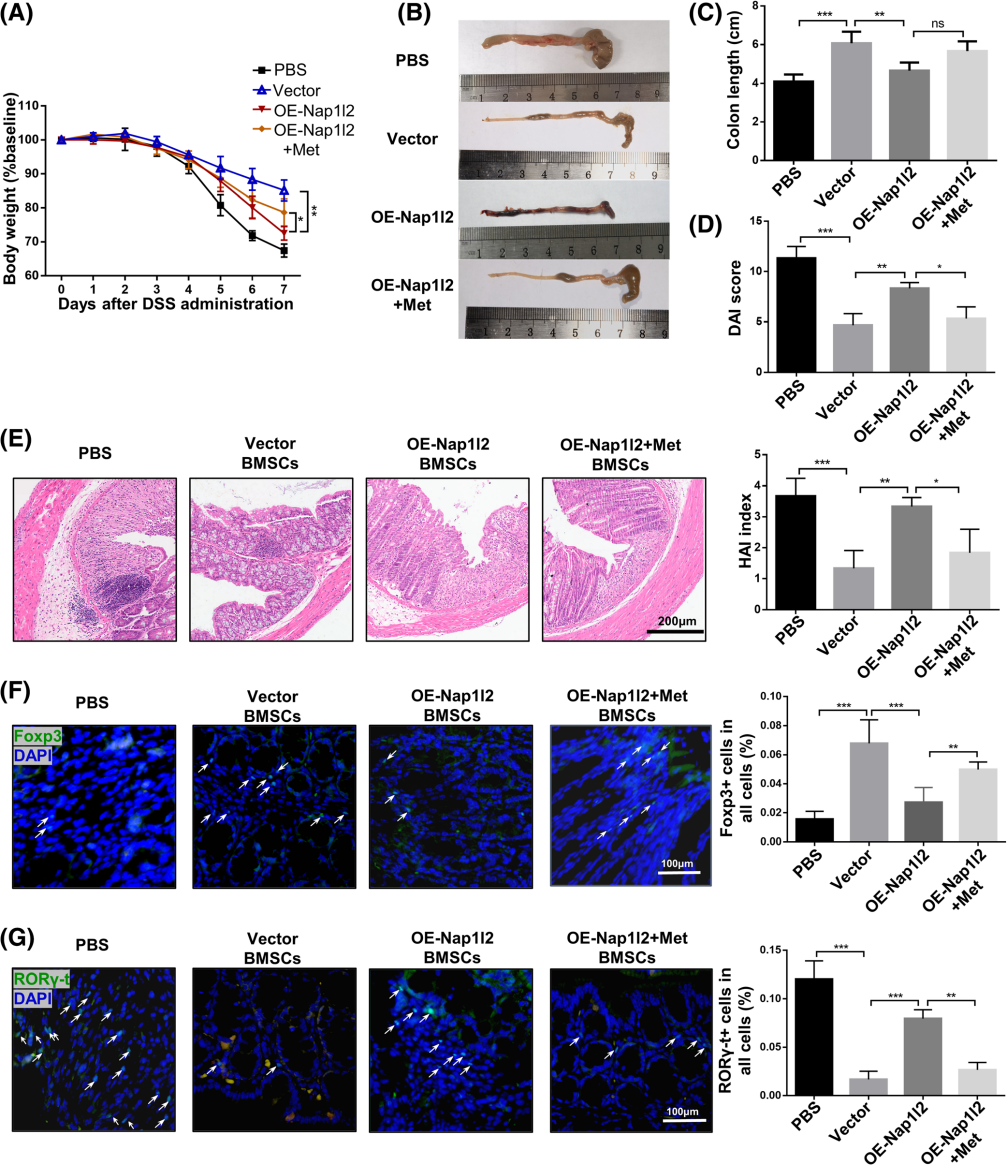
The effects of metformin on therapeutic efficiency of OE‐Nap1l2 BMSCs in DSS‐induced IBD. (A) Body weight, (B, C) colon length and (D) DAI of healthy mice and IBD mice treated with PBS, vector BMSCs, OE‐Nap1l2 BMSCs and metformin‐treated OE‐Nap1l2 BMSCs on day 1 and 4 post‐IBD induction. (E) HAI score and HE staining of colons. Scale bar, 200 μm. Levels of (F) Foxp3+ cells and (G) RORγ‐t + cells in situ (*white triangles*). Statistical significance was determined by one‐way ANOVA or two‐way ANOVA. Data were presented as mean ± SD (*n* ≥ 3). *p < 0.05, **p < 0.01, ***p < 0.001; ns, no significance.

Then, to explore the effect of metformin on OE‐Nap1l2 BMSCs, metformin‐treated OE‐Nap1l2 BMSCs were administered through tail vein injection in IBD and EAE mice. The results showed that in the IBD mice managed with metformin‐treated OE‐Nap1l2 BMSCs, weight loss was ameliorated, DAI was decreased, and shortening of the colon length was also alleviated (Figure 6A–D). The levels of Tnf‐α and Il17 mRNA in the colon were significantly decreased in the group injected with metformin‐treated OE‐Nap1l2 BMSCs (Figure S10A,B). H&E staining demonstrated less inflammatory infiltration and epithelial ulceration in the metformin‐treated OE‐Nap1l2 BMSCs group (Figure 6E). Compared with OE‐Nap1l2 BMSCs, the number of Tregs was increased and the number of Th17 cells was decreased in the metformin‐treated OE‐Nap1l2 BMSCs group (Figure 6F,G).

In EAE mice, the metformin‐treated OE‐Nap1l2 BMSCs group also exhibited a favourable outcome as compared with the OE‐Nap1l2 BMSCs group. The clinical symptoms were significantly alleviated and leukocyte infiltration was reduced in the spinal cord (Figure S11A,B). Taken together, metformin could promote the treatment efficacy of OE‐Nap1l2 BMSCs in autoimmune diseases.

## 4 DISCUSSION

In the present study, we demonstrated that Nap1l2 has significantly impaired the migration and immunomodulation capabilities of BMSCs. The efficacy of BMSCs was reduced after overexpression of Nap1l2 in treating with autoimmune diseases IBD and EAE in mouse models. However, we observed that metformin could enhance the immunoregulation ability of OE‐Nap1l2 BMSCs. Metformin was shown to affect the metabolism of OE‐Nap1l2 BMSCs, by increasing the ratio of AMP/ATP ratio and activating the AMPK signalling pathway. Furthermore, metformin activated immune modulation of BMSCs by increasing the generation of l‐arginine, stimulating iNOS expression, thereby leading to increased NO production.

MSCs senescence is a dynamic process in that gene expression profiles are modified with impaired properties of cell proliferation, multiple differentiation, homing ability, and immune modulation.^36^ Senescence cells perform SASP which features is preponderance of pro‐inflammatory molecules, leading to chronic inflammation and accumulation of senescence.^37^, ^38^, ^39^ Previous studies revealed that manipulation of Nap1l2 altered BMSCs senescence through the NF‐κB pathway. Nap1l2 can regulate DNA damage and the expression of SASP factor genes, p16, and p21 to manage the process of cellular senescence.^21^ In this study, the migration ability was suppressed after the overexpression of Nap1l2 but was alleviated after the knockdown of Nap1l2. Under the inflammatory microenvironment, high expression of Il‐1, Il‐6 and depressed expression of Il10, Tgf‐β was elicited after overexpression of Nap1l2, while the opposite outcomes were observed in BMSCs after knockdown of Nap1l2. These immune‐regulatory molecules are involved in the development, survival and function of various immune cells. TGFβ could induce foxp3 gene expression and mediate the transition of CD4 + CD25‐naïve T cells to a regulatory T cell phenotype,^40^, ^41^ but IL‐6 facilitates Th17 cell generation by reducing the positive effect of TGFβ on TGFβ type I receptor expression.^42^ Therefore, changes in the expression of these inflammatory factors suggested the impaired immunomodulatory ability of BMSCs.

Previously, MSCs were reported to have the capacity to modulate adaptive immunological responses by suppressing the proliferation of T cells and altering the phenotypes of the T cell population.^43^ Numerous studies have demonstrated that cell senescence suppresses the immunological regulation of MSCs.^44^, ^45^ BMSCs from elder mice have markedly weak regulation on macrophages, and the amount of M2 macrophages is significantly lessened when coculturing with senescent BMSCs.^46^ The growth of peripheral blood mononuclear cells is suppressed by MSCs that separate from young and elderly people, however, the elderly group is less affected.^18^ Our research revealed that OE‐Nap1l2 BMSCs failed to inhibit splenocyte proliferation when cocultured with spleen cells. In this study, we found that significantly less Tregs and more Th17 were induced by OE‐Nap1l2 BMSCs. In vivo, research demonstrated that Nap1l2 had a positive influence on the therapeutic efficacy of BMSCs. Moreover, within the inflammatory microenvironment, the immune modulation ability of mouse BMSCs is managed by the expression of iNOS and NO.^33^, ^47^, ^48^ Manipulation of Nap1l2 alters iNOS expression and arginine synthesis, which may affect the immunomodulation properties of MSCs through NO production.

Furthermore, KEGG pathway analysis suggested that the elevating of the AMP/ATP ratio is closely associated with the AMPK signalling pathway. Accumulating evidence has revealed that the metabolism regulator AMPK, which senses low‐energy states by detecting high levels of AMP, plays an important role in promoting longevity.^49^ Previous studies demonstrated a suppressed AMPK phosphorylation level in replicative senescence periodontal ligament stem cells. In our study, manipulation of Nap1l2 could affect the activation of the AMPK signalling pathway. Metformin is a common medicine for patients with type 2 diabetes, as it can inhibit complex I of the mitochondrial electron transport chain (ETC), thus activating the AMPK pathway by increasing the AMP/ATP ratio, thereby affecting the cell energy metabolism and protein synthesis.^50^ In our study, metformin affected the oxidative phosphorylation level by upregulating the AMP/ATP ratio, promoting AMPK phosphorylation, and activating the AMPK signalling pathway. Moreover, metformin could promote iNOS expression and arginine synthesis in BMSCs, as well as elevate NO generation, which enhanced the potential of mouse BMSCs to modulate the immune response.

In addition, metformin has been shown to have anti‐cancer, immunoregulatory and anti‐ageing effects. It can relieve ageing‐related inflammation by stabilizing the function of mitochondria,^51^, ^52^ and also affect the therapeutic efficacy of chronic disease‐induces MSCs through the AMPK signalling pathway.^53^ In our study, the migration ability of OE‐Nap1l2 BMSCs was facilitated by metformin, along with the change in the secretion of inflammatory factors. In a coculturing system of spleen cells and BMSCs, the proportion of immune cells was obviously altered in the metformin group, where a decreased number of Th17 cells and an increased number of Treg cells were observed. In vivo, analysis in mice revealed that the ability of OE‐Nap1l2 BMSCs to treat inflammatory diseases was rescued by metformin.

Taken together, we demonstrated that Nap1l2 had a negative effect on the immunomodulation capacities of BMSCs. Metformin treatment could rescue the immunomodulation capacities of BMSCs by activating the AMPK signalling pathway. The translational merit of our study is to provide metformin as an applicable pharmacological target for the rejuvenation of senescent BMSCs and for achieving higher efficacy in stem cell therapy.

## AUTHOR CONTRIBUTIONS

F.L., Y.C., X.Z. and F.G. contributed to performing the experiment, data acquisition and analysis; F.L., R.H., S.N. contributed to writing the manuscript; F.L., Z.W., L.X. and Z. O. contributed to the animal experiments; S.L., W.M., L.S. and X.W. contributed to experiment support, data analysis, manuscript discussion and revision; D.L., M.H. contributed to the conception of the study and critically reviewed the manuscript.

## FUNDING INFORMATION

This work was supported by the National Natural Science Foundation of China (82071079 to Dayong Liu). Jinmen Medical Talents Project of Tianjin Health Commission (TJSJMYXYC‐D2‐025 to Dayong Liu).

## CONFLICT OF INTEREST STATEMENT

The authors disclose no potential conflicts of interest.

## Supporting information


**Figure S1.** Nap1l2 impaired the migration and inflammatory cytokine production of BMSCs. (A) Quantitative RT–PCR showing expression of Nap1l2 in BMSCs after Nap1l2 overexpression or knockdown. (B) Representative images showing scratch assay of BMSCs with Nap1l2 overexpression or knockdown at 0, 24 and 48 h. The rate of migration was calculated by the width of the wound. Scale bar, 200 μm. (C) Transwell migration assay and quantitative analysis showing the migration abilities of BMSCs after Nap1l2 overexpression or knockdown. Scale bar, 200 μm. (D) Detecting the inflammatory cytokine Il1, Il6, Tgfβ and Il10 mRNA levels in BMSCs treated with TNF‐α, IFN‐γ (20 ng/mL each) for 24 h. Statistical significance was determined by one‐way ANOVA. Data were presented as mean ± SD (*n* ≥ 3). *p < 0.05, **p < 0.01, ***p < 0.001; ns, not significance.

**Figure S2.** Nap1l2 impaired the T cell regulation capacities of BMSCs. (A) The gating strategy of CD4+ T cells that were cocultured with BMSCs. (B) The gating strategy of CD3 + CD8 − IL17+ cells in splenocytes (Spl) cocultured with BMSCs after Nap1l2 overexpression or knockdown. (C)The gating strategy of CD4 + CD25 + Foxp3+ cells in splenocytes cocultured with BMSCs after Nap1l2 overexpression or knockdown.

**Figure S3.** (A, B) The expressions of pro‐inflammatory cytokines Il17 and Tnf‐α in the colon after injected with BMSCs. Statistical significance was determined by one‐way ANOVA.

**Figure S4.** The therapeutic effects of BMSCs in experimental autoimmune encephalomyelitis (EAE). (A) Clinical disease score of healthy mice and EAE mice treated with PBS, vector BMSCs, OE‐Nap1l2 BMSCs, NC BMSCs and KD‐Nap1l2 BMSCs. (B) Representative H&E staining of spinal cord sections from healthy mice and EAE mice at day 18 post MOG_35‐55_ immunization. Scale bar, 400 μm. Statistical significance was determined by two‐way ANOVA. Data were presented as mean ± SD (*n* ≥ 3). *p < 0.05, **p < 0.01, ***p < 0.001; ns, no significance.

**Figure S5.** Global metabolic profiling of BMSCs. (A) The OPLS–DA model showing differences among vector BMSCs and OE‐Nap1l2 BMSCs. (B) The permutation plot showing the stable and reliable of vector BMSCs and OE‐Nap1l2 BMSCs. (C) The OPLS–DA model showing the differences between OE‐Nap1l2 BMSCs and metformin‐treated OE‐Nap1l2 BMSCs. (D) The permutation plot showing the stable and reliable of OE‐Nap1l2 BMSCs and metformin‐treated OE‐Nap1l2 BMSCs. (E) The proportion of various metabolites. (F) The number of metabolites identified in the positive and negative ion modes, respectively (*n* = 6).

**Figure S6.** Energy metabolism characterization of BMSCs. (A–C) Heat map of (A) fatty acid metabolism, (B) glycolysis/gluconeogenesis and (C) amino acid metabolism of Vector BMSCs and OE‐Nap1l2 BMSCs. (D–F) Heat map of (D) fatty acid metabolism, (E) glycolysis/gluconeogenesis and (F) amino acid metabolism of OE‐Nap1l2 BMSCs and metformin‐treated OE‐Nap1l2 BMSCs (*n* = 6).

**Figure S7.** Depletion of Nap1l2 altered the metabolism of replicative senescence BMSCs. (A, B) β‐galactosidase staining and quantitative analysis of BMSCs at passage 4 (P4) and passage 16 (P16). (C) Quantitative RT–PCR analysis of the expression of senescence‐related genes *p16* and *p21*. (D) Quantitative RT–PCR showing expression of Nap1l2 in BMSCs after Nap1l2 knockdown. (E) Protein levels of iNOS in BMSCs after Nap1l2 knockdown. (F) Griess reagent assaying for nitrate from the supernatant of BMSCs after Nap1l2 knockdown. (G) Protein expression levels of p‐AMPK and AMPK after Nap1l2 knockdown. Statistical significance was determined by one‐way ANOVA. Data were presented as mean ± SD (*n* ≥ 3). *p < 0.05, **p < 0.01, ***p < 0.001; ns, not significance.

**Figure S8.** The effects of metformin on cell migration and inflammatory cytokine secretion of OE‐Nap1l2 BMSCs. (A) Representative images and quantitative assay showing scratch assay of BMSCs with Nap1l2 overexpression and treated with 100 μM metformin for 24 h. Scale bar, 200 μm. (B) Transwell migration assay and quantitative analysis showing the increased migration capacity of OE‐BMSCs after being treated with 100 μM metformin for 24 h. Scale bar, 200 μm. (C) Quantitative RT–PCR analysis of the expression of Il1, Il6, Tgfβ and Il10 in TNF‐α, IFN‐γ stimulated BMSCs. Statistical significance was determined by one‐way ANOVA. Data were presented as mean ± SD (*n* ≥ 3). *p < 0.05, **p < 0.01, ***p < 0.001; ns, no significance.

**Figure S9.** The effects of metformin on OE‐Nap1l2 BMSCs in T cell subsets regulation. (A) The gating strategy of CD4+ T cells that were cocultured with BMSCs. (B) The gating strategy of CD3 + CD8 − IL17+ cells in splenocytes (Spl) cocultured with BMSCs. (C) The gating strategy of CD4 + CD25 + Foxp3+ cells in splenocytes cocultured with BMSCs.

**Figure S10.** (A, B) The expressions of pro‐inflammatory cytokines Tnf‐α and Il17 in the colon after injected with BMSCs. Statistical significance was determined by one‐way ANOVA.

**Figure S11.** Metformin improved the therapeutic efficiency of Nap1l2 overexpression BMSCs in EAE. (A) Clinical disease score of healthy mice and EAE mice treated with PBS, Vector BMSCs, OE‐Nap1l2 BMSCs and metformin‐treated OE‐Nap1l2 BMSCs. (B) Representative H&E staining of spinal cord sections. Statistical significance was determined by two‐way ANOVA. Scale bar, 400 μm. Data were presented as mean ± SD (*n* ≥ 3). *p < 0.05, **p < 0.01, ***p < 0.001; ns, no significance.

**Table S1.** Key Materials of this study.

**Table S2.** Primers used in this study.

**Data S1.** Supporting information.

## Data Availability

The data sets used and analysed during the current study are available from the corresponding author upon reasonable request.
