# Supplementary figures and images for: Metformin rejuvenates Nap1l2‐impaired immunomodulation of bone marrow mesenchymal stem cells via metabolic reprogramming
Source: Cell Prolif. 2024 Feb 13;57(7):e13612. doi: 10.1111/cpr.13612 (PMC11216924; doi:10.1111/cpr.13612)

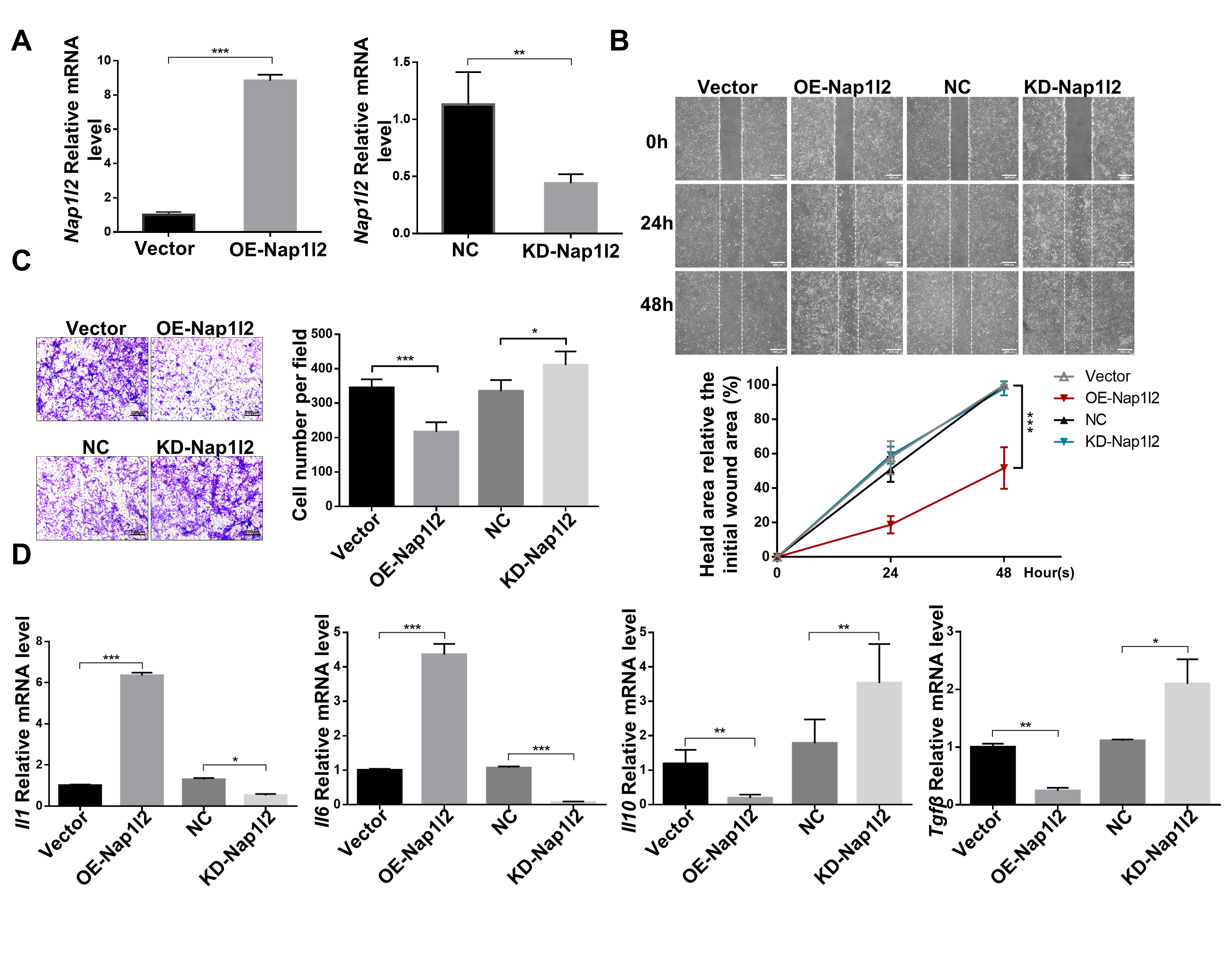

Supplement: Supplementary file 1 — Figure S1. Nap1l2 impaired the migration and inflammatory cytokine production of BMSCs. (A) Quantitative RT–PCR showing expression of Nap1l2 in BMSCs after Nap1l2 overexpression or knockdown. (B) Representative images showing scratch assay of BMSCs with Nap1l2 overexpression or knockdown at 0, 24 and 48 h. The rate of migration was calculated by the width of the wound. Scale bar, 200 μm. (C) Transwell migration assay and quantitative analysis showing the migration abilities of BMSCs after Nap1l2 overexpression or knockdown. Scale bar, 200 μm. (D) Detecting the inflammatory cytokine Il1, Il6, Tgfβ and Il10 mRNA levels in BMSCs treated with TNF‐α, IFN‐γ (20 ng/mL each) for 24 h. Statistical significance was determined by one‐way ANOVA. Data were presented as mean ± SD (n ≥ 3). *p < 0.05, **p < 0.01, ***p < 0.001; ns, not significance. [file CPR-57-e13612-s006.tif]

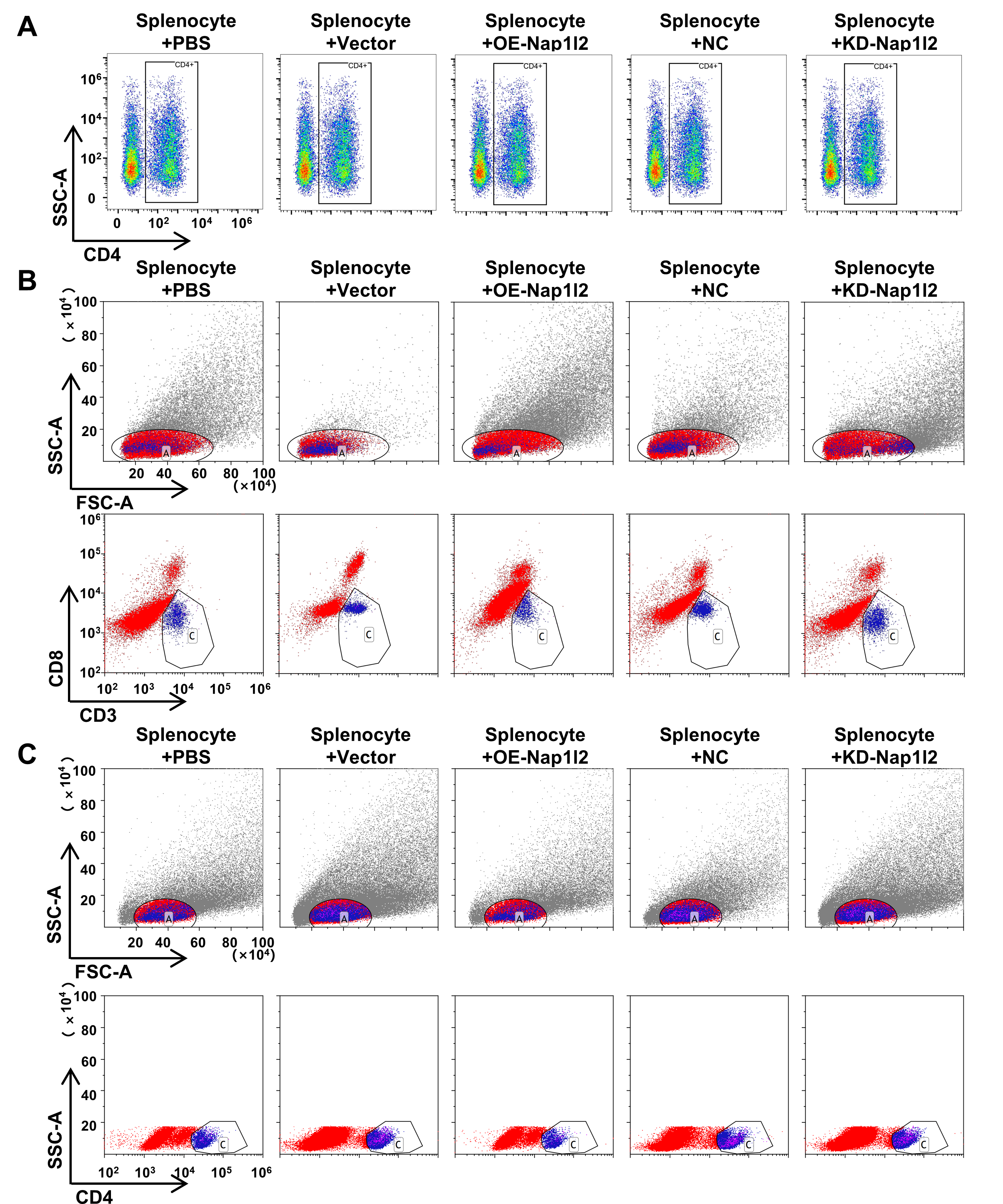

Supplement: Supplementary file 2 — Figure S2. Nap1l2 impaired the T cell regulation capacities of BMSCs. (A) The gating strategy of CD4+ T cells that were cocultured with BMSCs. (B) The gating strategy of CD3 + CD8 − IL17+ cells in splenocytes (Spl) cocultured with BMSCs after Nap1l2 overexpression or knockdown. (C)The gating strategy of CD4 + CD25 + Foxp3+ cells in splenocytes cocultured with BMSCs after Nap1l2 overexpression or knockdown. [file CPR-57-e13612-s007.tif]

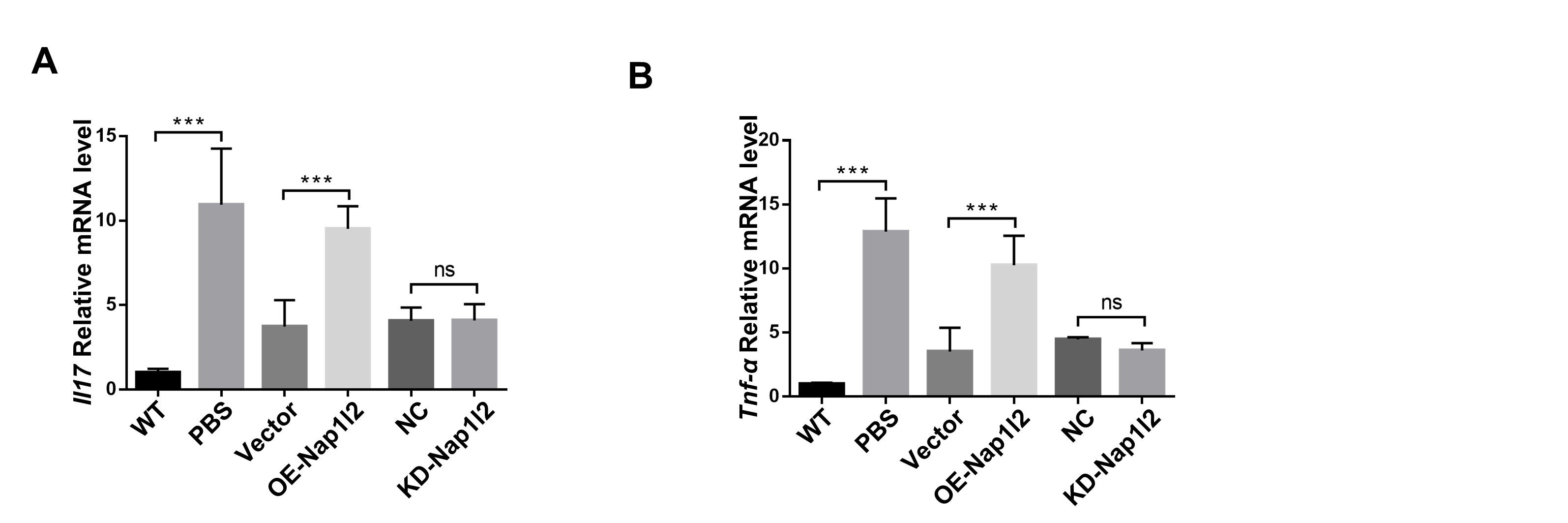

Supplement: Supplementary file 3 — Figure S3. (A, B) The expressions of pro‐inflammatory cytokines Il17 and Tnf‐α in the colon after injected with BMSCs. Statistical significance was determined by one‐way ANOVA. [file CPR-57-e13612-s009.tif]

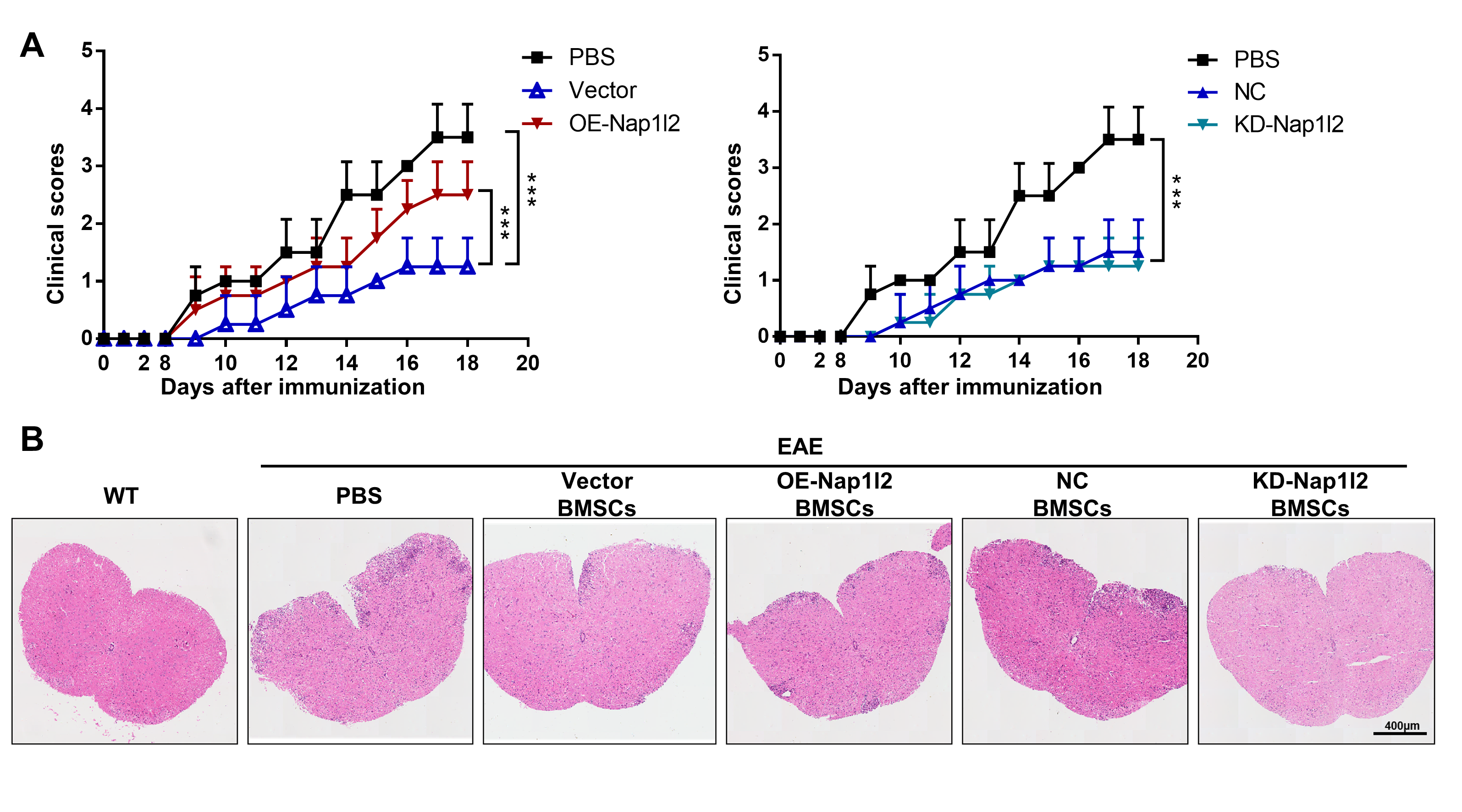

Supplement: Supplementary file 4 — Figure S4. The therapeutic effects of BMSCs in experimental autoimmune encephalomyelitis (EAE). (A) Clinical disease score of healthy mice and EAE mice treated with PBS, vector BMSCs, OE‐Nap1l2 BMSCs, NC BMSCs and KD‐Nap1l2 BMSCs. (B) Representative H&E staining of spinal cord sections from healthy mice and EAE mice at day 18 post MOG35‐55 immunization. Scale bar, 400 μm. Statistical significance was determined by two‐way ANOVA. Data were presented as mean ± SD (n ≥ 3). *p < 0.05, **p < 0.01, ***p < 0.001; ns, no significance. [file CPR-57-e13612-s012.tif]

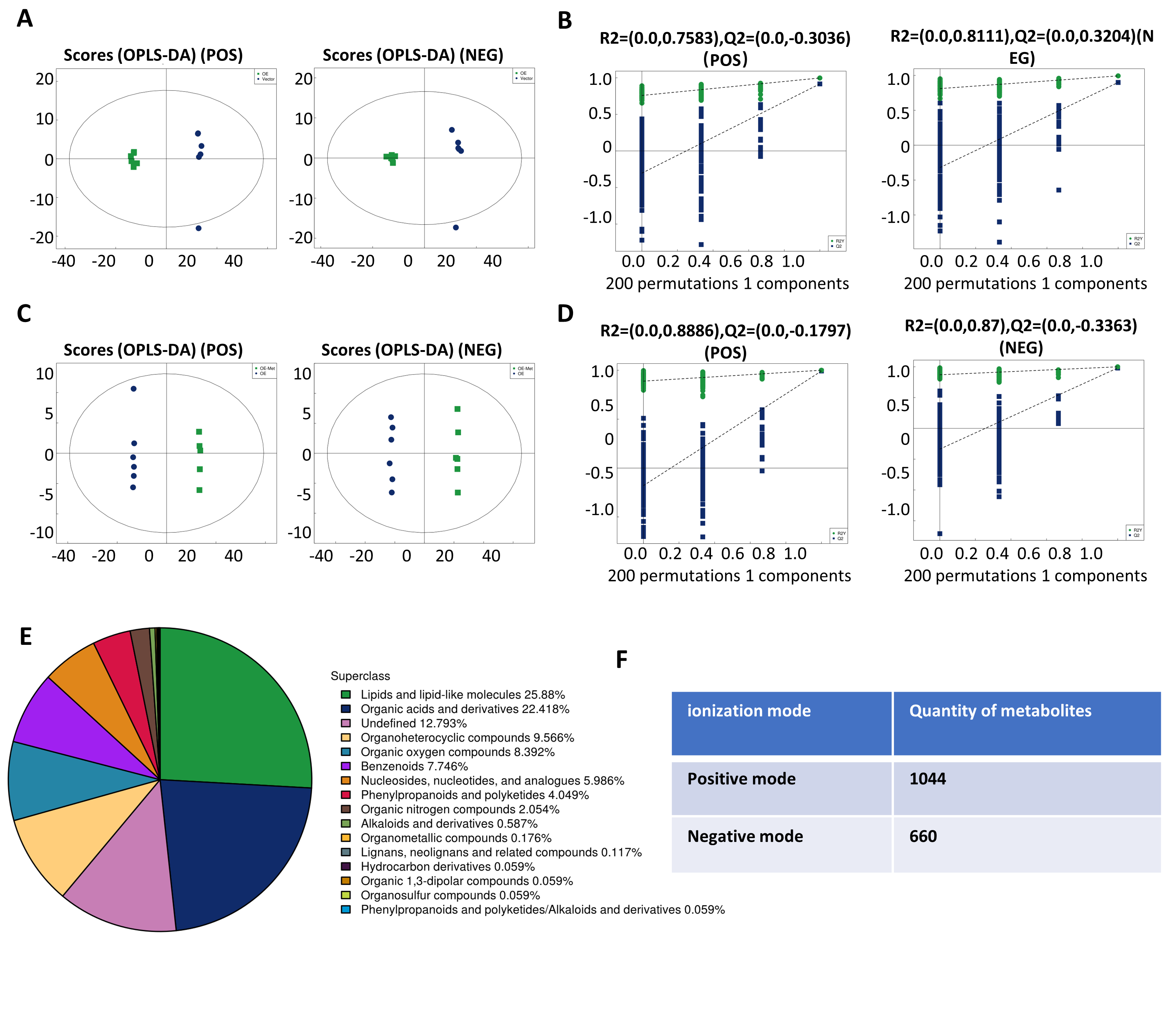

Supplement: Supplementary file 5 — Figure S5. Global metabolic profiling of BMSCs. (A) The OPLS–DA model showing differences among vector BMSCs and OE‐Nap1l2 BMSCs. (B) The permutation plot showing the stable and reliable of vector BMSCs and OE‐Nap1l2 BMSCs. (C) The OPLS–DA model showing the differences between OE‐Nap1l2 BMSCs and metformin‐treated OE‐Nap1l2 BMSCs. (D) The permutation plot showing the stable and reliable of OE‐Nap1l2 BMSCs and metformin‐treated OE‐Nap1l2 BMSCs. (E) The proportion of various metabolites. (F) The number of metabolites identified in the positive and negative ion modes, respectively (n = 6). [file CPR-57-e13612-s004.tif]

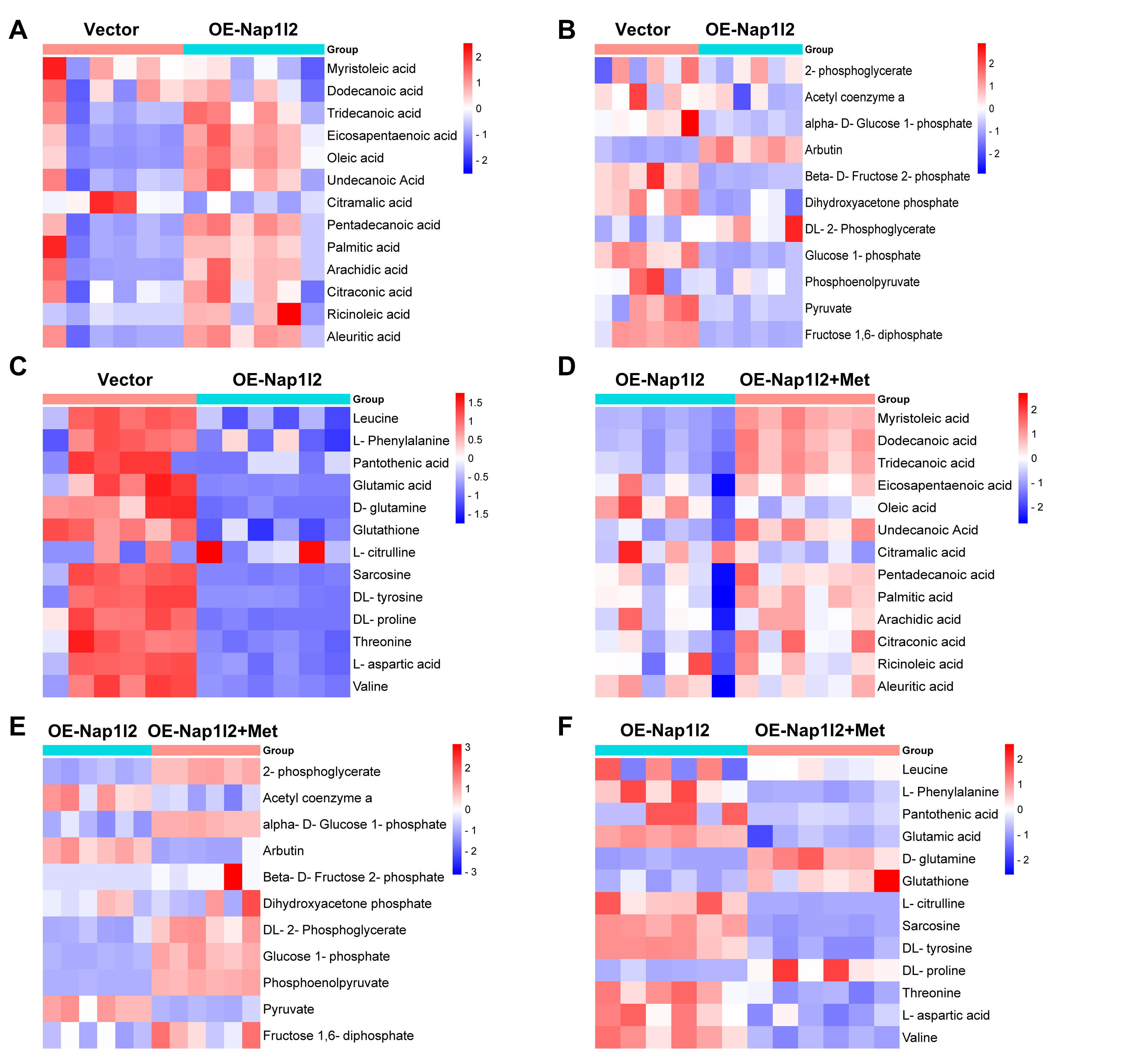

Supplement: Supplementary file 6 — Figure S6. Energy metabolism characterization of BMSCs. (A–C) Heat map of (A) fatty acid metabolism, (B) glycolysis/gluconeogenesis and (C) amino acid metabolism of Vector BMSCs and OE‐Nap1l2 BMSCs. (D–F) Heat map of (D) fatty acid metabolism, (E) glycolysis/gluconeogenesis and (F) amino acid metabolism of OE‐Nap1l2 BMSCs and metformin‐treated OE‐Nap1l2 BMSCs (n = 6). [file CPR-57-e13612-s001.tif]

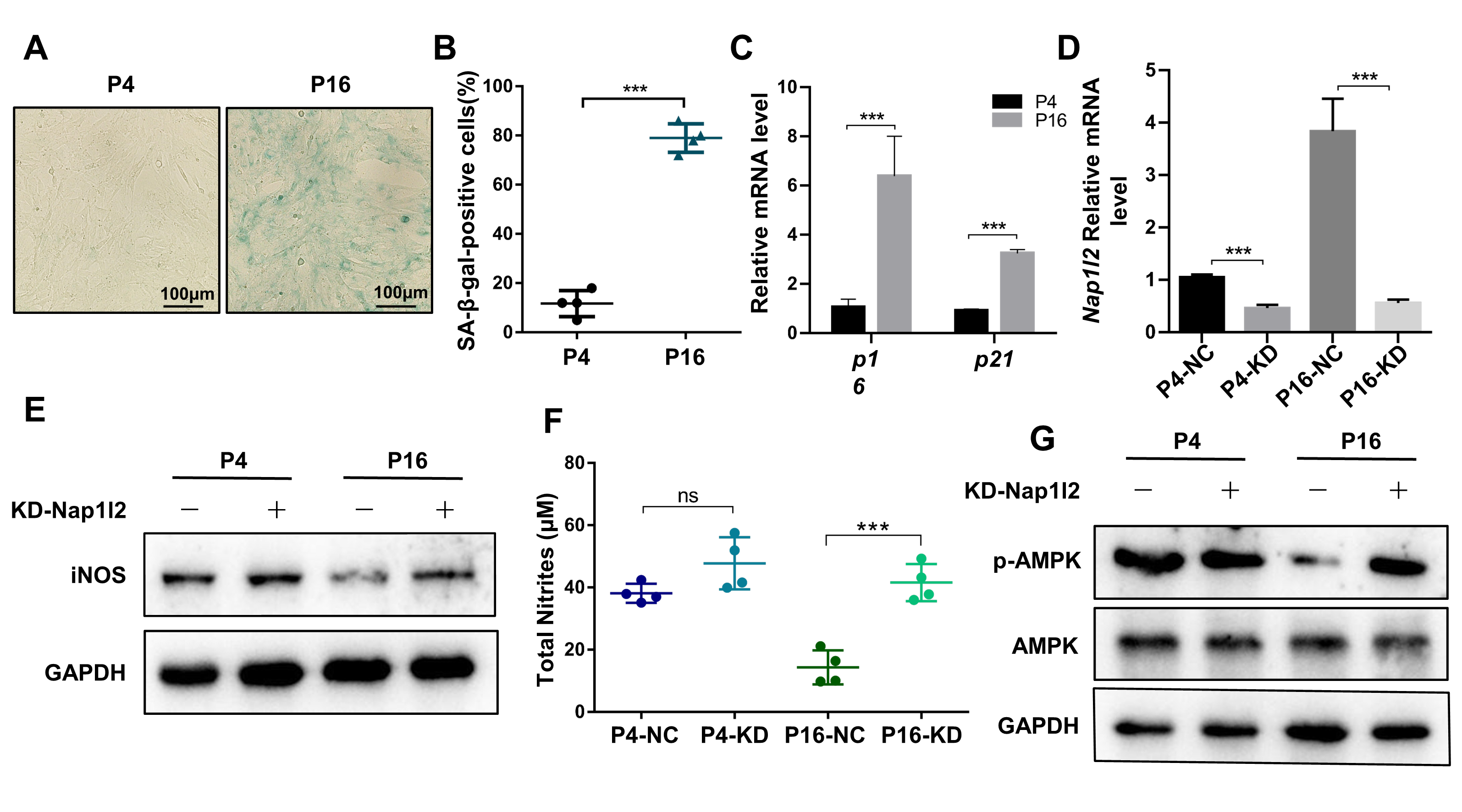

Supplement: Supplementary file 7 — Figure S7. Depletion of Nap1l2 altered the metabolism of replicative senescence BMSCs. (A, B) β‐galactosidase staining and quantitative analysis of BMSCs at passage 4 (P4) and passage 16 (P16). (C) Quantitative RT–PCR analysis of the expression of senescence‐related genes p16 and p21. (D) Quantitative RT–PCR showing expression of Nap1l2 in BMSCs after Nap1l2 knockdown. (E) Protein levels of iNOS in BMSCs after Nap1l2 knockdown. (F) Griess reagent assaying for nitrate from the supernatant of BMSCs after Nap1l2 knockdown. (G) Protein expression levels of p‐AMPK and AMPK after Nap1l2 knockdown. Statistical significance was determined by one‐way ANOVA. Data were presented as mean ± SD (n ≥ 3). *p < 0.05, **p < 0.01, ***p < 0.001; ns, not significance. [file CPR-57-e13612-s011.tif]

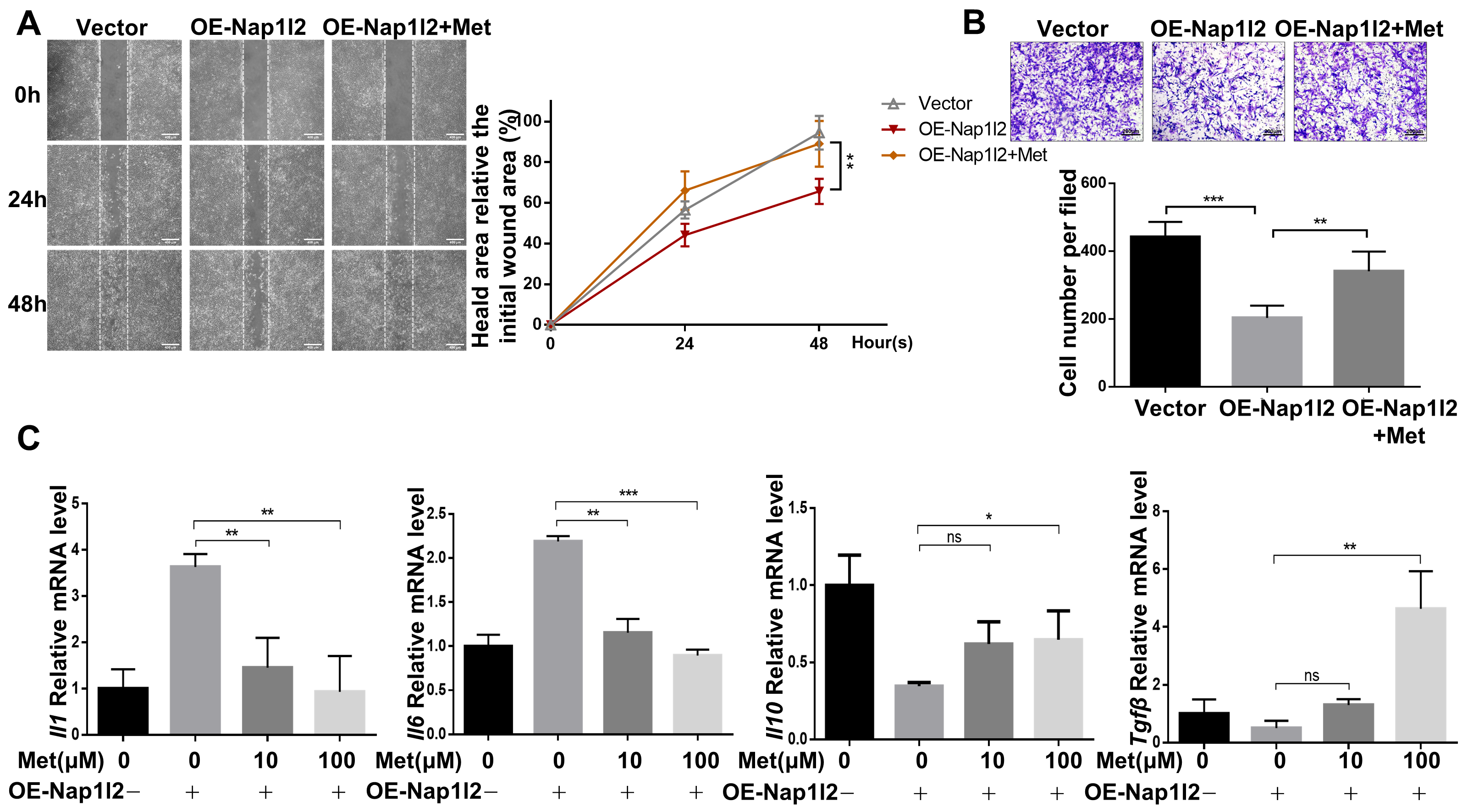

Supplement: Supplementary file 8 — Figure S8. The effects of metformin on cell migration and inflammatory cytokine secretion of OE‐Nap1l2 BMSCs. (A) Representative images and quantitative assay showing scratch assay of BMSCs with Nap1l2 overexpression and treated with 100 μM metformin for 24 h. Scale bar, 200 μm. (B) Transwell migration assay and quantitative analysis showing the increased migration capacity of OE‐BMSCs after being treated with 100 μM metformin for 24 h. Scale bar, 200 μm. (C) Quantitative RT–PCR analysis of the expression of Il1, Il6, Tgfβ and Il10 in TNF‐α, IFN‐γ stimulated BMSCs. Statistical significance was determined by one‐way ANOVA. Data were presented as mean ± SD (n ≥ 3). *p < 0.05, **p < 0.01, ***p < 0.001; ns, no significance. [file CPR-57-e13612-s014.tif]

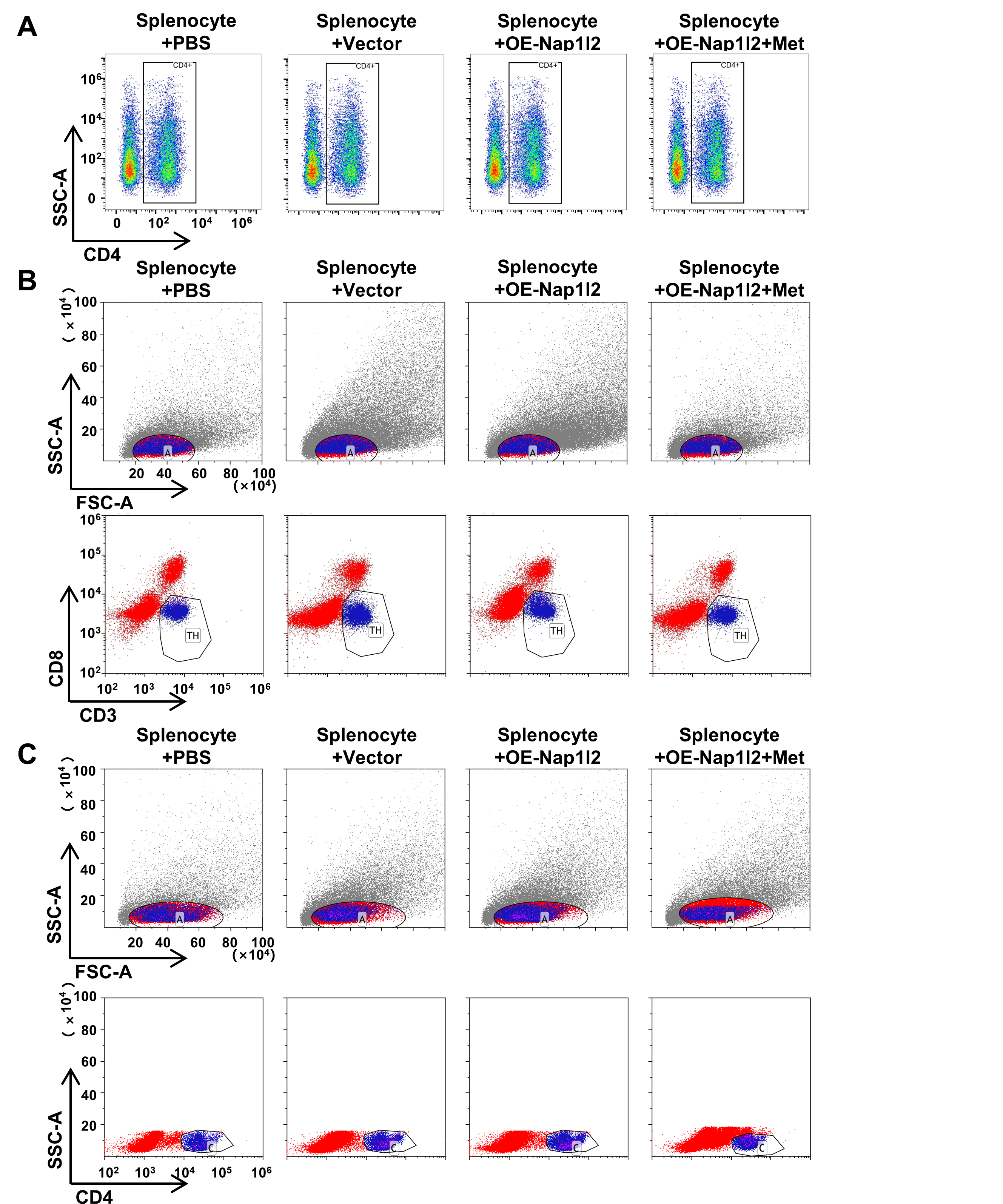

Supplement: Supplementary file 9 — Figure S9. The effects of metformin on OE‐Nap1l2 BMSCs in T cell subsets regulation. (A) The gating strategy of CD4+ T cells that were cocultured with BMSCs. (B) The gating strategy of CD3 + CD8 − IL17+ cells in splenocytes (Spl) cocultured with BMSCs. (C) The gating strategy of CD4 + CD25 + Foxp3+ cells in splenocytes cocultured with BMSCs. [file CPR-57-e13612-s005.tif]

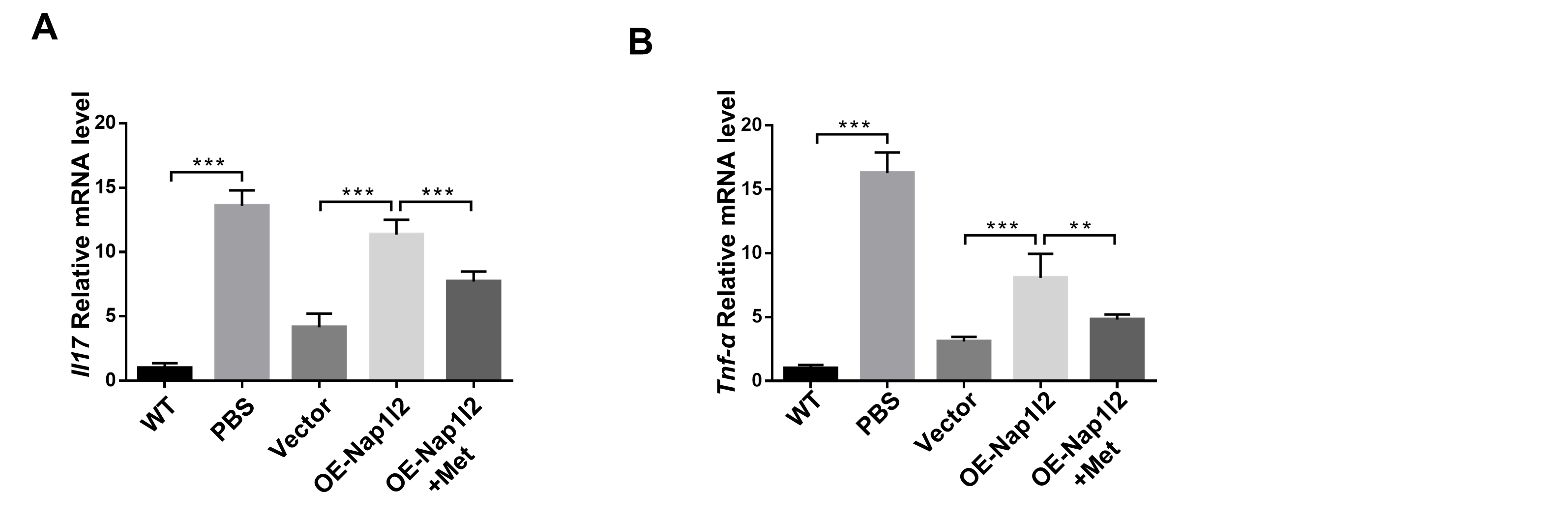

Supplement: Supplementary file 10 — Figure S10. (A, B) The expressions of pro‐inflammatory cytokines Tnf‐α and Il17 in the colon after injected with BMSCs. Statistical significance was determined by one‐way ANOVA. [file CPR-57-e13612-s010.tif]

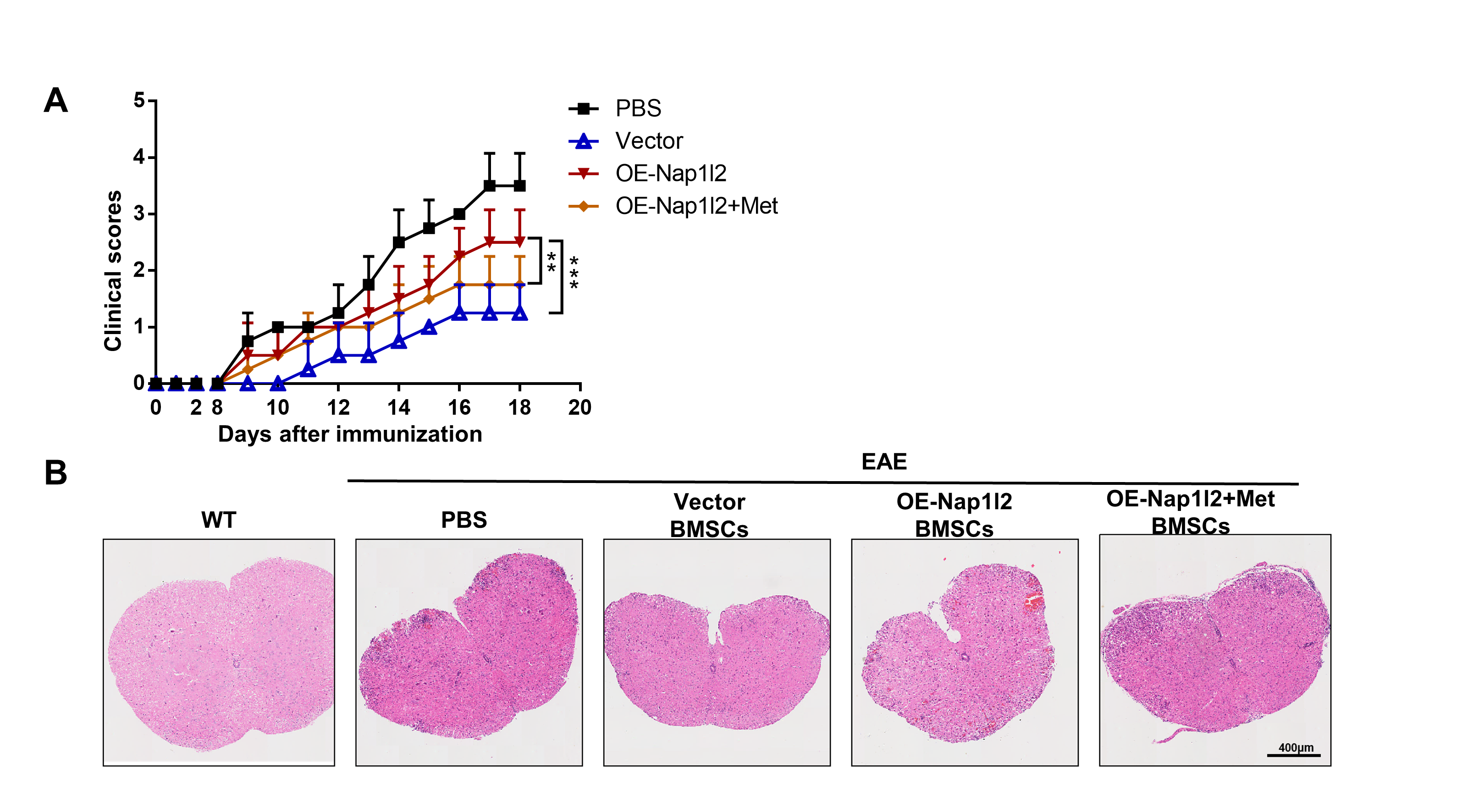

Supplement: Supplementary file 11 — Figure S11. Metformin improved the therapeutic efficiency of Nap1l2 overexpression BMSCs in EAE. (A) Clinical disease score of healthy mice and EAE mice treated with PBS, Vector BMSCs, OE‐Nap1l2 BMSCs and metformin‐treated OE‐Nap1l2 BMSCs. (B) Representative H&E staining of spinal cord sections. Statistical significance was determined by two‐way ANOVA. Scale bar, 400 μm. Data were presented as mean ± SD (n ≥ 3). *p < 0.05, **p < 0.01, ***p < 0.001; ns, no significance. [file CPR-57-e13612-s002.tif]
