# Supplementary material for: Metformin rejuvenates Nap1l2‐impaired immunomodulation of bone marrow mesenchymal stem cells via metabolic reprogramming
Source: Cell Prolif. 2024 Feb 13;57(7):e13612. doi: 10.1111/cpr.13612 (PMC11216924; doi:10.1111/cpr.13612)
Supplement: Supplementary file 12 — Table S1. Key Materials of this study. [file CPR-57-e13612-s013.docx]

**Supplementary Table 1. Key Materials of this study**

| **Category** | **Source** | **Cat. No.** |
| --- | --- | --- |
| **Antibodies** | | |
| FITC anti-mouse CD8a | Biolegend | 100705 |
| FITC anti-mouse CD4 | Biolegend | 116003 |
| PE anti-mouse CD25 | Biolegend | 101903 |
| PerCP/Cyanine5.5 anti-mouse CD3 | Biolegend | 100217 |
| PE/Cyanine7 anti-mouse IL-17A | Biolegend | 506921 |
| Alexa Fluor 647 anti-mouse FOXP3 | Biolegend | 320013 |
| Anti-AMPK rabbit | Cell signaling technology | 2535T |
| Anti-p-AMPK rabbit | Cell signaling technology | 5831T |
| Anti-GAPDH rabbit | Abcam | ab181602 |
| Anti-iNOS rabbit | Abmart | T55993S |
| Goat Anti-Rabbit IgG-HRP | Sigma-Aldrich | A0545 |
| Anti-rabbit IgG | Proteintech | 30000-0-AP |
| **Drugs** | | |
| Metformin | Sigma | PHR1084-500MG |
| Dynabeads® Mouse T-Activator CD3/CD28 | Gibco | 11452D |
| Recombinant Mouse IL-2 | Biolegend | 575404 |
| Cell Stimulation Cocktail (500X) | TonboBio | TNB-4975-UL100 |
| CellTrace^TM^ CFSE Cell Proliferation Kit | ThermoFisher Scientific | C34554 |
| Tuberculosis H37 Ra, Desiccated | BD | 231141 |
| Incomplete Freund’s adjuvant | Sigma | F5506 |
| Pertussis toxin, Lyophilized Salt-Free | List biological laboratories | 181 |
| Myelin oligodendrocyte glycoprotein  _35-55_ | Nanjing Peptide Biotech Ltd. | NJP64618 |
| Dextran Sulfate Sodium Salt | MP Biomedicals | 021601180 |
| **Critical Commercial Assays** | | |
| HiScript III RT SuperMix for qPCR | Vazyme | R323-01 |
| Universal SYBR qPCR Master Mix | Vazyme | Q711 |
| Pierce BCA Protein Assay Kit | ThermoFisher Scientific | 23225 |
| Phosphatase Inhibitor Cocktail(100×) | Cell Signaling technology | 5871S |
| TRIzol Reagent | Ambion, Life Science | 15596018 |
| Total Nitric Oxide Assay Kit | Beyotime | S0023 |
| CellTrace CFSE Cell Proliferation Kit | Invitrogen | C34554 |
