# Supplementary material for: Metformin rejuvenates Nap1l2‐impaired immunomodulation of bone marrow mesenchymal stem cells via metabolic reprogramming
Source: Cell Prolif. 2024 Feb 13;57(7):e13612. doi: 10.1111/cpr.13612 (PMC11216924; doi:10.1111/cpr.13612)
Supplement: Supplementary file 13 — Table S2. Primers used in this study. [file CPR-57-e13612-s008.docx]

**Supplementary Table 2. Primers used in this study.**

| **Primers sequence** | |
| --- | --- |
| *Gapdh-F* | TCAAGCTCATTTCCTGGTATGACA |
| *Gapdh-R* | TAGGGCCTCTCTTGCTCAGT |
| *Tnf-α-F* | TATGGCCCAGACCCTCACA |
| *Tnf-α-R* | GGAGTAGACAAGGTACAACCCATC |
| *Il1-F* | AAGGAGAACCAAGCAACGACAAA |
| *Il1-R* | TGGGGAACTCTGCAGACTCAAACT |
| *iNOS-F* | CAAGCTGAACTTGAGCGAGGA |
| *iNOS-R* | TTTACTCAGTGCCAGAAGCTGGA |
| *Il10-F* | GCCAGAGCCACATGCTCCTA |
| *Il10-R* | GATAAGGCTTGGCAACCCAAGTAA |
| *Tgfβ-F* | TGATACGCCTGAGTGGCTGTCT |
| *Tgfβ-R* | CACAAGAGCAGTGAGCGCTGAA |
