# Supplementary material for: Metformin rejuvenates Nap1l2‐impaired immunomodulation of bone marrow mesenchymal stem cells via metabolic reprogramming
Source: Cell Prolif. 2024 Feb 13;57(7):e13612. doi: 10.1111/cpr.13612 (PMC11216924; doi:10.1111/cpr.13612)
Supplement: Supplementary file 14 — Data S1. Supporting information. [file CPR-57-e13612-s003.docx]

Supplementary Material

**Metformin rejuvenates Nap1l2-impared immunomodulation of bone marrow mesenchymal stem cells via metabolic reprogramming**

**Fan Liu^1^, Ruohui Han ^1^, Shaochen Nie ^1^, Yuxin Cao^1^, Xinming Zhang^1^, Feng Gao ^1^, Zhengyang Wang ^1^, Liangyu Xing ^1^, Zhaoguang Ouyang ^1^, Lei Sui ^2^, Wenyi Mi ^3^, Xudong Wu ^4^, Lu Sun^5，6^, Meilin Hu ^1*^, Dayong Liu^1*^**

**^*^ Correspondence:** Dayong Liu, DDS., Ph.D., Professor, Department of Endodontics and Laboratory of Stem Cells Endocrine Immunology, Tianjin Medical University School of Stomatology, 12 Qixiangtai Road, Tianjin 300070, China Tel./Fax: +86 2223332096, E-Mail: dyliuperio@tmu.edu.cn or

Meilin Hu, DDS, Ph.D., Tianjin Medical University School of Stomatology, 12 Qixiangtai Road, Tianjin 300070, China. E-Mail, humeilin@tmu.edu.cn

**Supplementary methods and materials**

**SA-β-GAL staining**

Cellular senescence was assessed using the β-galactosidase staining kit, following the manufacturer’s instructions (Beyotime, China). Cells were washed 3 times with PBS, and fixed with β-galactosidase staining fixative for 15 min. After washing with PBS, added staining working solution and incubated the 6-well plate overnight at 37°C by sealing with parafilm. The samples were observed under an ordinary optical microscope.

**EAE Induction**

To induce EAE in mice, 400 ug MOG_35-55_ (Nanjing Peptide Biotech Ltd., China) in complete Freund’s adjuvant containing M. Tuberculosis (BD, USA) was inoculated subcutaneously on the back. Intravenous administration of 250 ng Pertussis toxin (List biological laboratories, China) was performed immediately and 48 hours later. For EAE mice, caudal vein injection of BMSCs was managed on day 9, 11, and 13. The drugs used in this study are listed in Supplementary Table 1.

**Supplementary Figures Legends**

**Supplementary Figure 1.** Nap1l2 impaired the migration and inflammatory cytokine production of BMSCs. (**A**) Quantitative RT-PCR showing expression of *Nap1l2* in BMSCs after Nap1l2 overexpression or knockdown. (**B**) Representative images showing scratch assay of BMSCs with Nap1l2 overexpression or knockdown at 0 h,24 h and 48 h. The rate of migration was calculated by the width of the wound. Scale bar, 200μm. (**C**) Transwell migration assay and quantitative analysis showing the migration abilities of BMSCs after Nap1l2 overexpression or knockdown. Scale bar, 200μm. (**D**) Detecting the inflammatory cytokine *Il1*, *Il6*, *Tgfβ* and *Il10* mRNA levels in BMSCs treated with TNF-α, IFN-γ (20 ng/mL each) for 24 h. Statistical significance was determined by one-way ANOVA. Data were presented as mean ± SD (n ≥ 3). **p* < 0.05, ***p* < 0.01, ****p* < 0.001; ns, not significance.

**Supplementary Figure 2.** Nap1l2 impared the T cell regulation capacities of BMSCs. (**A**) The gating strategy of CD4+ T cells that were cocultured with BMSCs. (**B**) The gating strategy of CD3+CD8−IL17+ cells in splenocytes (Spl) cocultured with BMSCs after Nap1l2 overexpression or knockdown. (**C**)The gating strategy of CD4+CD25+Foxp3+ cells in splenocytes cocultured with BMSCs after Nap1l2 overexpression or knockdown.

**Supplementary Figure 3.** (**A**-**B**)The expressions of pro-inflammatory cytokines *Il17* and *Tnf-α* in colon after injected with BMSCs. Statistical significance was determined by one-way ANOVA.

**Supplementary Figure 4.** The therapeutic effects of BMSCs in experimental autoimmune encephalomyelitis (EAE). (**A**) Clinical disease score of healthy mice and EAE mice treated with PBS, vector BMSCs, OE-Nap1l2 BMSCs, NC BMSCs and KD-Nap1l2 BMSCs. (**B**) Representative H&E staining of spinal cord sections from healthy mice and EAE mice at day 18 post MOG_35-55_ immunization. Scale bar, 400μm. Statistical significance was determined by two-way ANOVA. Data were presented as mean ± SD (n≥3). *p < 0.05, **p < 0.01, ***p < 0.001; ns, no significance.

**Supplementary Figure 5.** Global metabolic profiling of BMSCs. (**A**) The OPLS-DA model showing differences among vector BMSCs and OE-Nap1l2 BMSCs. (**B**) The permutation plot showing the stable and reliable of vector BMSCs and OE-Nap1l2 BMSCs. (**C**) The OPLS-DA model showing the differences of OE-Nap1l2 BMSCs and metformin treated OE-Nap1l2 BMSCs. (**D**) The permutation plot showing the stable and reliable of OE-Nap1l2 BMSCs and metformin treated OE-Nap1l2 BMSCs. (**E**) The proportion of various metabolites. (**F**) The number of metabolites identified in the positive and negative ion mode, respectively. (n=6)

**Supplementary Figure 6.** Energy metabolism characterization of BMSCs. (**A-C)** Heat map of (**A**) fatty acid metabolism, (**B**) glycolysis/gluconeogenesis and (**C**) amino acid metabolism of Vector BMSCs and OE-Nap1l2 BMSCs. (**D-F**) Heat map of (**D**) fatty acid metabolism, (**E**) glycolysis/gluconeogenesis and (**F**) amino acid metabolism of OE-Nap1l2 BMSCs and metformin treated OE-Nap1l2 BMSCs. (n=6)

**Supplementary Figure 7.** Depletion of Nap1l2 altered he metabolism of replicative senescence BMSCs. (**A**-**B**) β-galactosidase staining and quantitative analysis of BMSCs at passage 4 (P4) and passage 16 (P16). (**C**) Quantitative RT-PCR analysis of the expression of senescence-related gene *p16* and *p21*. (**D**) Quantitative RT-PCR showing expression of *Nap1l2* in BMSCs after Nap1l2 knockdown. (**E**) Protein levels of iNOS in BMSCs after Nap1l2 knockdown. (**F**) Griess reagent assaying for nitrate from the supernatant of BMSCs after Nap1l2 knockdown. (**G**) Protein expression levels of p-AMPK and AMPK after Nap1l2 knockdown. Statistical significance was determined by one-way ANOVA. Data were presented as mean ± SD (n ≥ 3). *p < 0.05, **p < 0.01, ***p < 0.001; ns, not significance.

**Supplementary Figure 8.** The effects of metformin on cell migration and inflammatory cytokine secretion of OE-Nap1l2 BMSCs. (**A**) Representative images and quantitative assay showing scratch assay of BMSCs with Nap1l2 overexpression and treated with 100μM metformin for 24h. Scale bar, 200 μm. (**B**) Transwell migration assay and quantitative analysis showing the increased migration capacity of OE-BMSCs after treated with 100 μM metformin for 24 h. Scale bar, 200 μm. (**C**) Quantitative RT-PCR analysis of the expression of *Il1*, *Il6*, *Tgfβ* and *Il10* in TNF-α, IFN-γ stimulated BMSCs. Statistical significance was determined by one-way ANOVA. Data were presented as mean ± SD (n ≥ 3). **p* < 0.05, ***p* < 0.01, ****p* < 0.001; ns, no significance.

**Supplementary Figure 9.** The effects of metformin on OE-Nap1l2 BMSCs in T cell subsets regulation. (**A**) The gating strategy of CD4+ T cells that were cocultured with BMSCs. (**B**) The gating strategy of CD3+CD8−IL17+ cells in splenocytes (Spl) cocultured with BMSCs. (**C**)The gating strategy of CD4+CD25+Foxp3+ cells in splenocytes cocultured with BMSCs .

**Supplementary Figure 10.** (**A**-**B**)The expressions of pro-inflammatory cytokines *Tnf-α* and *Il17* in colon after injected with BMSCs. Statistical significance was determined by one-way ANOVA.

**Supplementary Figure 11.** Metformin improved the therapeutic efficiency of Nap1l2 overexpression BMSCs in EAE.(**A**) Clinical disease score of healthy mice and EAE mice treated with PBS, Vector BMSCs, OE-Nap1l2 BMSCs and metformin treated OE-Nap1l2 BMSCs. (**B**) Representative H&E staining of spinal cord sections. Statistical significance was determined by two-way ANOVA. Scale bar, 400μm. Data were presented as mean ± SD (n≥3). *p < 0.05, **p < 0.01, ***p < 0.001; ns, no significance.
